# Oncogenic TRIB2 interacts with and regulates PKM2 to promote aerobic glycolysis and lung cancer cell procession

**DOI:** 10.1038/s41420-022-01095-1

**Published:** 2022-07-05

**Authors:** Yuan-Rong Liu, Dan-Dan Song, Dong-Min Liang, You-Jie Li, Yun-Fei Yan, Hong-Fang Sun, Mei-Ling Zhang, Jin-Xia Hu, Yu-Long Zhao, Yan Liang, Yan-Mei Li, Zhen Yang, Ran-Ran Wang, Hou-Feng Zheng, Pingyu Wang, Shu-Yang Xie

**Affiliations:** 1 Department of Biochemistry and Molecular Biology, Binzhou Medical University, 264003 YanTai, Shandong PR China; 2 Department of Physiology and Pathophysiology, School of Basic Medicine, Qingdao University, 266071 QingDao, Shandong PR China; 3 Department of Immune Rheumatism, Yantaishan Hospital, 264000 Yantai, Shandong PR China; 4 Institute of Rehabilitation Medicine, School of Rehabilitation Medicine, Binzhou Medical University, 264003 Yantai, Shandong PR China; 5 School of Life Sciences, Westlake University, Hangzhou, 310024 Zhejiang, China; 6 Department of Epidemiology, Binzhou Medical University, 264003 YanTai, ShanDong PR China

**Keywords:** Cancer metabolism, Targeted therapies

## Abstract

PKM2 is an important regulator of the aerobic glycolysis that plays a vital role in cancer cell metabolic reprogramming. In general, *Trib2* is considered as a “pseudokinase”, contributing to different kinds of cancer. However, the detailed roles of TRIB2 in regulating cancer metabolism by PKM2 remain unclear. This study demonstrated that TRIB2, not a “pseudokinase”, has the kinase activity to directly phosphorylate PKM2 at serine 37 in cancer cells. The elevated pSer37-PKM2 would subsequently promote the PKM2 dimers to enter into nucleus and increase the expression of LDHA, GLUT1, and PTBP1. The aerobic glycolysis is then elevated to promote cancer cell proliferation and migration in TRIB2- or PKM2-overexpressed cultures. The glucose uptake and lactate production increased, but the ATP content decreased in TRIB2- or PKM2-treated cultures. Experiments of TRIB2^−/−^ mice further supported that TRIB2 could regulate aerobic glycolysis by PKM2. Thus, these results reveal the new kinase activity of TRIB2 and its mechanism in cancer metabolism may be related to regulating PKM2 to promote lung cancer cell proliferation in vitro and in vivo, suggesting promising therapeutic targets for cancer therapy by controlling cancer metabolism.

## Introduction

Alteration in cell metabolism is a hallmark of many cancers and contributes to cancer cell proliferation, survival and migration [1]. The increased consumption of glucose but decreased production of ATP was defined as “Warburg effect”, which enables cancer cells to acquire and metabolize nutrients in favor of proliferation rather than efficient ATP production [2, 3]. Lung cancer is one of the most common carcinomas worldwide, and the proteomics and metabonomics studies have screened out many proteins playing vital roles in the high heterogeneity of cell metabolic pathways [4].

The M2 isoform of pyruvate kinase (PKM2) elevated in lung, breast, cervix, kidney, bladder, papillary thyroid, colon, and prostate cancer [5]. PKM2 is encoded by *Pkm*2 (15q23) gene, an important regulator of the Warburg effect that plays a central role in cancer cell metabolic reprogramming [6] and increases cell glucose utilization and alterations in the redox balance. PKM2 actually exists as inactive monomer, less active dimer, and active tetramer. In contrast to the high capacity of tetramer in ATP production, PKM2 dimer promotes the conversion of glucose-derived pyruvate to lactate through lactate dehydrogenase [7]. In addition to low PK activity, PKM2 dimer also has the “non-glycolysis enzyme function” of entering the nucleus as a transcription factor to activate the transcription of certain genes and integrating with other transcription factors to regulate gene transcription [8]. These genes include the aerobic glycolysis-related glucose transporter 1 (GLUT1), lactate dehydrogenase A (LDHA) [9], a hypoxia inducible factor-1α, β-catenin, insulin, and others that promote cell growth and proliferation [10, 11]. The most common event to upregulate PKM2 dimer is the phosphorylation of PKM2 at Ser 37, which promotes PKM2 to translocate to the nucleus [12]. Hence, pSer37-PKM2 is important for the dimer forms of PKM2 and the regulation of its related genes. However, whether PKM2 Ser 37 could be phosphorylated by other factors remains to be elucidated.

Tribbles (TRIBs) were first identified as a crucial cell cycle regulator in Drosophila [13] and include three mammalian homologues: Trib1, Trib2, and Trib3. TRIBs family functions as scaffolding molecules to help protein degradation via a proteasome–dependent mechanism [14]. *Trib2* gene is first identified as a myeloid oncogene, which contributes to acute myeloid leukemia (AML) in a bone marrow transplant model [15]. The oncogenic roles of TRIB2 have also been described in other cancers where it is overexpressed, such as lung cancer [16], liver cancer[17], melanoma [18], and pancreatic cancer [19]. In addition, TRIB2 overexpression can affect the sensitivity of cancer cells to anti-cancer drugs [20]. Through its interaction with different factors like CDC25, MAPK, OCT3/4, AP4, ubiquitin E3 ligases, C/EBP alpha, AKT, and MAPK, TRIB2 plays an important roles in cellular processes, such as senescence, cell cycle, protein degradation, as well as cell survival [20]. The functions of TRIB2 are closely related to its structure and can be divided into 3 parts: an N-terminal domain (PEST), a C-terminal E3 ligase-binding domain, and a pseudokinase domain with a Ser/Thr protein kinase-like domain [21]. Owing to its importance in tumorigenesis and therapeutic resistance, targeting TRIB2 may present an exciting opportunity for cancer therapy and anti-cancer drug design. To date, the detailed roles of TRIB2 in cancer and the function of “pseudokinase domain” remain unclear.

In the last decade, investigation on the roles of TRIB2 in lung cancer found that some microRNAs like miR-511, miR-1297 and let-7c can effectively inhibit lung cancer proliferation by suppressing the expression of TRIB2 and consequently increasing that of C/EBPα [22, 23]. Via reduced phospho-Smad3/Smad3, miR-206 and miR-140 downregulate TRIB2 to further suppress lung cancer cell proliferation and metastasis [24]. In the present work, the affinity purification and mass spectrometry were employed to analyze the TRIB2 interactome in vitro to further investigate the detailed mechanism of TRIB2 and its interacting with protein in lung cancer. Results demonstrated that TRIB2 could interact with many biochemical metabolism-related proteins, including PKM2. TRIB2 also has the kinase activity to directly phosphorylate PKM2 at serine 37 in cancer cells, and the elevated pSer37-PKM2 would promote the dimers to enter into nucleus and to increase the aerobic glycolysis in cancer cells.

## Materials and methods

### Human NSCLC tissues

Sectioned non-small cell lung cancer (NSCLC) tissues were collected between January 1, 2018 and December 30, 2020 from the Inpatient Department of Chest surgery, Yantai Shan Hospital, the Teaching Hospital of Binzhou Medical University (Yantai, China). This study included 49 patients (30 males and 19 females, aged 35–65 years) pathologically diagnosed with NSCLC for the first time and had not yet received chemotherapy. Fresh NSCLC tissues and para-carcinoma controls from the patients who underwent surgery were also collected and examined. The levels of p-PKM2 (ser37)/PKM2 and TRIB2 in the tissues were analyzed to investigate their roles in lung cancer. All experiments were approved and performed in accordance with the Medical Ethics Committee of Binzhou Medical University. The study procedures were fully explained to patients prior to study inclusion, and the patients provided written informed consent.

### Cell cultures

The human BEAS-2B, A549, H1299, H1975, HeLa, and 293T cell lines were obtained from the Shanghai Institute of Cell Biology, China. Cells were cultured in a standard humidified incubator with 5% CO_2_ at 37 °C, with RPMI-1640 medium (Gibco, Grand Island, New York, USA) supplemented with 10% fetal bovine serum (FBS, Gibco). The cell lines have been detected without mycoplasma contamination.

### GST pull-down assay and mass spectrometry assay

GST pull-down assays were performed as previously reported [25]. GST-fusion proteins were induced to express by 0.5 mM IPTG in Escherichia coli (BL21) for over 20 h. The bacteria were collected and sonicated in lysis buffer. After the recipitates were removed from the lysates, nickel beads (QIAGEN China, Shanghai, China) or glutathione sepharose beads (Amersham Pharmacia) were incubated with the supernatants for 4 h at 4 °C. The beads were collected after three times washing with lysis buffer, and GST-fusion proteins were obtained from the beads. The GST-TRIB2 pull-down proteins were subjected to Coomassie brilliant blue staining and analyzed by mass spectrometry (Q Exactive LC-MS/MS, Thermo, Germany). Cell lysates from A549, H1299, or other cells were incubated with GST-fusion protein bound to GST beads for 4 h at 4 °C and the adsorbed proteins were further analyzed by immunoblotting.

### Co-IP

Co-Immunoprecipitation(Co-IP) was performed as previously described [26]. Total lysate was extracted with lysis buffer (50 mM Tris-Cl at pH 7.4, 1 mM EDTA, 150 mM NaCl, 0.5% SDS, 0.5% NP-40, protease inhibitor mixture) and incubated with Anti-Flag M2 Affinity Gel (Sigma–Aldrich; Darmstadt, Germany). Following centrifugation, the supernatant was subjected to SDS-PAGE, followed by immunoblotting. For the examination of endogenous PKM2-TRIB2 interaction, the cell lysates were incubated with primary antibodies or control IgG overnight at 4 °C. At the next day, the lysates were incubated with protein G/A beads (Invitrogen, Carlsbad, CA, USA) for 2 h at 4 °C. The beads were washed and mixed with protein loading buffer, and detected via immunoblotting.

### Immunoblotting

Immunoblotting was performed as previously described [24]. The cells were harvested and lysed by lysis buffer (P0013, Beyotime Biotechnology, China), and the proteins were separated on SDS-PAGE gels and transferred onto PVDF membranes. The membranes were then incubated with rabbit anti-human p-PKM2(Ser37)/PKM2 (1:500; SignalwayAntibody LLC, Maryland, USA), rabbit anti-human TRIB2 (1:800, CST Ltd., USA), mouse anti-human CyclinD1 (1:500, Bioss Ltd., Boston, USA), rabbit anti-human OCT4 (1:500) and Lamin B1(1:1000, Proteintech Ltd., Manchester, UK), rabbit anti-human GLUT1 (1:500), LDHA (1:500), PTBP1 (1:800), HIF-1α(1:1000), c-Myc (1:500), α-tublin (1:1000), and β-actin (1:3000) (all from Bioworld Technology, Ltd., MN, USA) in TBST at 4 °C overnight. HRP-labeled goat anti-rabbit or mouse IgG (1:6000, Beijing Zhong Shan-Golden Bridge Technology Co., Ltd., Beijing, China) was added for 1 h incubation at room temperature. Immunoblotting was performed with ECL (Boster Immunoleader, Wuhan, China) and protein bands were quantified with Image J (NIH, Bethesda, MD, USA).

### Affinity measurement for interaction between TRIB2 and PKM2

Interaction between TRIB2 and PKM2 was analyzed by using BLI (ForteBio Inc., Menlo Park, CA, USA). First, the recombinant TRIB2 protein (TP301210, OriGene, MD, USA) was biotinylated and loaded onto the SSA biosensors, which then blocked using biocytin (5 μg/ml) for 60 s. Diluted PKM2 (ab89364, Abcam, St Louis, MO, USA) in PBS was then added onto the SSA biosensors loaded with TRIB2. The real-time binding response (Δλ in nanometer, nm) and the kinetic parameters/affinities were calculated using Octet data analysis software.

### Phosphorylated kinase activity analysis

Protein TRIB2 (400 ng, TP301210, OriGene), PKM2 (400 ng, ab89364, Abcam), 1 μl of ATP (2pM, PV3227, Thermo Fisher Scientific, USA), and 3 μl of 10×buffer (200 mM tris-HCl, 500 mM KCl, 100 mM MgCl2) were added water to 30 μl reaction solution. The mixture was then bathed at 30 °C for 1.5 h, added 7.5 μl of 5×SDS loading buffer, denaturated at 98 °C for 10 min, and performed for immunoblotting.

### Immunofluorescence staining analyzed by a confocal microscope

Tumor cells seeded on glass coverslips were washed with ice-cold PBS, fixed in 4% paraformaldehyde for 15 min, and permeabilized with 0.1% NP-40 for 5 min. Following treatment with 10% normal goat serum (with 1% BSA) for 60 min, the cells were incubated with rabbit anti-human p37-PKM2 (1:400; Catalog No: #12822, Signalway Antibody LLC, USA) overnight at 4 °C. After three washes with PBS, the cells were then incubated with Alexa Fluor 594 donkey anti-mouse IgG (H + L) (Molecular probes, Eugene, OR, USA) at 37 °C for 1 h. Immunofluorescence was examined under a confocal microscope (LEICA TCS SPE, Leica, Dresden, Germany).

### Glucose uptake

The glucose uptake of cultured cells was measured by using Amplex^®^ Red Glucose/Glucose Oxidase Assay Kit (No.A22189, Invitrogen, USA). Glucose oxidase reacted with d-glucose to form d-gluconolactone and H2O2. In the presence of HRP, H2O2 reacted with the Amplex^®^ Red reagent to generate the red-fluorescent oxidation product, resorufin, which was detected at 560 nm in an ELISA reader (Multiskan FC, Thermo Fisher Scientific, Boston, MA, USA).

### Lactate measurement

Lactate was measured by using Lactate Assay Kit (Catalog Number KA0833, Taipei City). Lactate specifically reacted with an enzyme mix to generate a product. Lactate probe was then added to produce color (OD 570 nm), which was detected by an ELISA reader (Multiskan FC, Thermo Fisher Scientific).

### ATP content

ATP production was estimated using ATP bioluminescent somatic cell assay kit in accordance with the manufacturer’s instructions (No. FLASC, Sigma, St Louis, MO, USA). The kit was employed for the bioluminescent determination of ATP released from a suspension of viable somatic cells.

### MTT assay

3-(4,5-dimethylthiazol-2-yl)-2,5-diphenyltetrazolium bromide (MTT) assay was performed to estimate cell proliferation [24, 27]. Cells (1 × 10^4^) in each well of a 96-well flat bottom microtiter plate were transfected with siRNAs or plasmids for 48 h. At 4 h before the end of incubation, 10 μL of MTT (5 mg/mL, Sigma, MO, USA) was added to each well. The supernatant was removed, 100 μL DMSO (Sigma) was added, and the optical density (OD) was detected (570 nm) in an ELISA reader (Multiskan FC, Thermo Fisher Scientific).

### Cell migration

A total of 1 × 10^4^ cells per well were cultured in the top chamber of a CIM plate, and real-time detection of cell migration was performed on a RTCA station (xCELLigence System, Roche, Mannheim, Germany) to investigate the dynamic changes of cell migration.

### Lung cancer cell xenografts

All animal experiments were performed in accordance with the Guidelines for Care and Use of Laboratory Animals of National Institutes of Health guidelines and approved by the Committee on the Ethics of Animal Experiments of Binzhou Medical University. Animals were randomly grouped by using random number table.

Lentiviral-mediated siRNA, or TRIB2/PKM2-overexpressed vectors were constructed and produced as previously reported [28]. The fragment harboring H1 promoter and shRNA sequence was cloned into blunt-ended PacI-digested FUGW vector (kindly provided by Dr. Zack Wang, Massachusetts General Hospital, Harvard University). NheI–XbaI element containing TRIB2/PKM2 was inserted into the XbaI site of FUGW vector to form the overexpression vector. 293T cells were cultured for lentivirus production. In brief, 1 mg of viral vector, 0.9 mg of the appropriate gag/pol expression vector (Δ8.9), and 0.1 mg of VSVG expression vector were mixed to transfect 293T cells. Virus supernatant was harvested, filtered, and added to the recipient cells according to standard protocols. A549 cells treated with lentivirus stably expressing shRNA-TRIB2, siRNA-PKM2, TRIB2 or PKM2, were harvested. Totally 2 × 10^6^ cells were injected subcutaneously into the backs of male BALB/C-nude mice aged 6–8 weeks (HFK Bio-Technology, Beijing, China). The primary tumors were measured daily by a caliper. After 1 month, the animals were euthanized by intraperitoneally injecting a barbiturate.

### TRIB2^−/−^ mice model

TRIB2^−/−^ mice were prepared by knocking out the exon 2 of Trib2 gene (ENSMUST00000020922) with CRISPR/CAS9 technology (Najing Biomedical Research Institute, Nanjing, China) to further investigate the roles of TRIB2 in regulating the metabolism of glucose uptake, lactate production, and ATP content. The count of erythrocyte, thrombocyte, and immunocytes from wild type and TRIB2^−/−^ mice were analyzed with Blood Analysis System (XS-500i, Japan).

The bone marrow cells from TRIB2^−/−^ and wild type mice were cultured to investigate glucose uptake, lactate production, and ATP content. These cells from TRIB2^−/−^ mice were further treated with lv-TRIB2 lentivirus or control to detect the above testing contents. The mice were euthanized and immersed in 75% ethanol. Tissues were removed from legs with scissors and dissected away from body. The bones were then immersed in 75% ethanol for 5 min. After each end of bone was cut off, a 27 g needle/1 ml syringe was filled with DMEM (Gibco). Bone marrow was then expelled from both ends of the bone directed into a 15 ml cell culture dish. Bone marrow cells were transferred to 1.5 ml EP tube and centrifuged at 800 g/min, 10 min. After the supernatant was discarded, 100 μL PBS was added to resuspend cell precipitation, and 300 μL red blood cell lysate (RT122, TIANGEN BIOTECH CO.,LTD., Beijing, China) was added for 5 min, which was then centrifuged at 800 g/min, 10 min. The collected bone marrow cells were resuspended in high glucose DMEM with 15% FBS and cultured in an incubator with 5% CO_2_ at 37 °C.

### Statistics

SPSS 22.0 software (IBM Corp., Armonk, NY, USA) was used to analyze statistical significance. Normally distributed data were presented as mean ± SD. Student’s *t* test and ANOVA were used to compare two averages and multiple groups, respectively. For in homogeneous variance assumption, LSD test or Games–Howell test was employed to compare the means. Abnormally distributed data were presented as median (interquartile range), and Mann–Whitney U test and Kruskal–Wallis H test were used to compare two groups and multiple groups, respectively. Pearson’s correlation was applied to analyze the association between the variables. Chi-square test was utilized to compare the rates of two groups. Kaplan–Meier survival analysis was performed to analyze the relationship between the survival of patients with lung cancer and TRIB2 or PKM2 expression. Data of TRIB2 and PKM2 expression in lung adenocarcinoma from Oncomine or TCGA database were analyzed by Pearson’s correlation. *p* < 0.05 was considered as statistically significant difference.

## Results

### TRIB2 and PKM2 were elevated in patient tissues

**Fig. 1:**
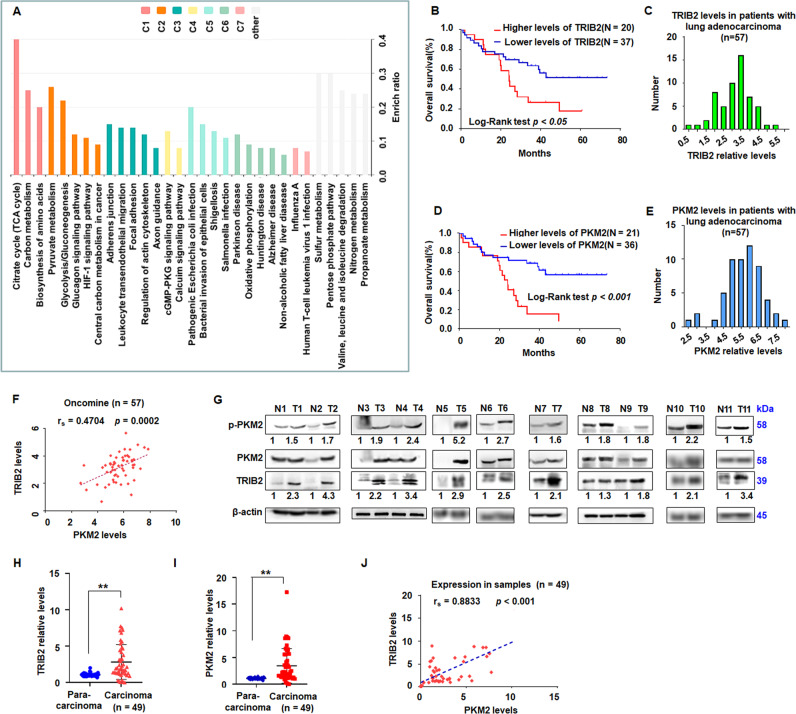
TRIB2 and PKM2 were overexpressed in the tissues of patients with lung cancer. **A** Gene enrichment analysis showing KEGG pathways involved in citrate cycle, carbon and pyruvate metabolism, and glycolysis. **B** Kaplan–Meier survival analysis indicated that high TRIB2 expression is associated with poor survival of patients with lung cancer, *p* < *0.05*. **C** TRIB2 levels in patients with lung adenocarcinoma. **D** Kaplan–Meier survival analysis showed that PKM2 expression is associated with poor survival of patients with lung cancer, *p* < *0.0001*. **E** PKM2 levels in patients with lung adenocarcinoma. **F** Data of TRIB2 and PKM2 expression in lung adenocarcinoma from Oncomine database and Pearson’s correlation (*n* = 57, **p = 0.0002). **G** Total proteins from 11 paired samples of lung carcinoma (T) versus adjacent normal tissues (N) were analyzed by immunoblotting. **H** qRT-PCR analysis of TRIB2 expression. Data were shown as median (interquartile range), **p < 0.01; *n* = 49, Mann–Whitney *U* test. **I** qRT-PCR analysis of PKM2 levels. Data were expressed as median (interquartile range), **p < 0.01; *n* = 49, Mann–Whitney *U* test. **J** Pearson’s correlation analysis of TRIB2 expression and PKM2 expression (*n* = 49, **p = 0.009).

Our previous studies supported that TRIB2 played an oncogenic role in tumorigenesis of lung cancer [23]. Here, TRIB2 expression increased in lung cancer (Fig. S1A), and GST-TRIB2-full were then constructed and incubated with the lysis of lung cancer tissues (Fig. S1B). The affinity purification and mass spectrometry were further employed to examine the TRIB2 interactome and investigate the mechanism of TRIB2 in lung cancer. The results showed that many proteins with high score related to biochemical metabolism, including PKM2, could interact with TRIB2 (Fig. 1A and Table S1).

Kaplan–Meier survival analysis of the data from Oncomine database (https://www.oncomine.org/resource/login.html) [24] indicated that poor survival for patients with high TRIB2 (*p* < 0.05, *n* = 57, (Fig. 1B, C) or high PKM2 levels (*p* < 0.001, *n* = 57, Fig. 1D, E). Moreover, TRIB2 were positively correlated with PKM2 levels (*p* = 0.0002, *n* = 57, Fig. 1F). Moreover, compared with those in lung para-carcinoma samples, the levels of TRIB2, PKM2, and p-PKM2 were elevated in lung carcinoma samples (Fig. 1G). qRT-PCR results showed that the expression of TRIB2 and PKM2 was higher in lung carcinoma samples than that in controls (*p* < 0.01, Fig. 1H, I), and TRIB2 was positively correlated with PKM2 expression (*p* = 0.009, Fig. 1J). Data from TCGA database further supported that TRIB2 and PKM2 expression was higher in lung carcinoma tissues (*p* < 0.01) (Fig. S1C, D).

### TRIB2 and PKM2 interact with each other

**Fig. 2:**
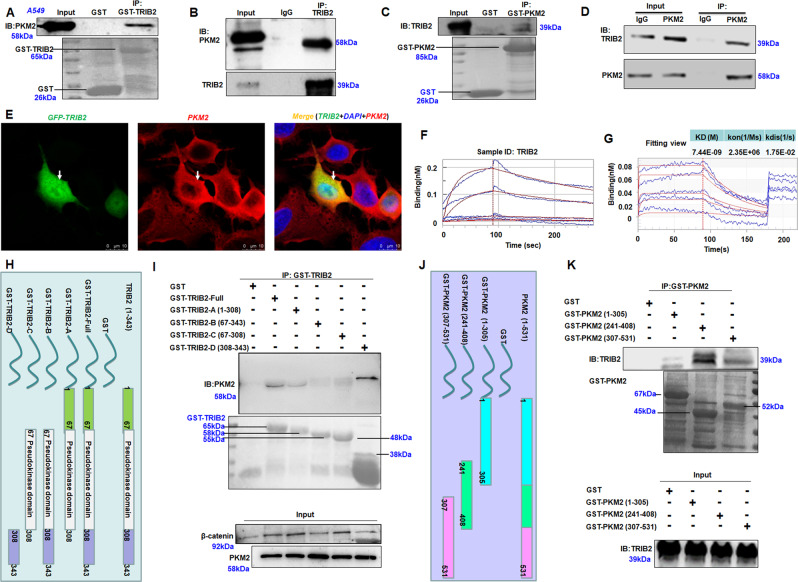
Analyzing and mapping of the interaction between TRIB2 and PKM2. **A** GST-TRIB2 was induced by IPTG and stably expressed in vitro (lower), and co-precipitation experiments incubated with lysates of A549 cells. **B** Co-IP with TRIB2 antibodies showing the interaction between TRIB2 and PKM2 in A549 cells. **C** GST-PKM2 expression was induced by IPTG in vitro (lower), and co-precipitation experiments incubated with lysates of A549 cells. **D** Co-IP with PKM2 antibodies showing the interaction between PKM2 and TRIB2 in A549 cells. **E** Co-localization of PKM2 (red) and GFP-TRIB2 (green) in A549 cells, observed via confocal microscopy. Scale bar = 10 μm. Arrow indicates the co-localization of PKM2 and GFP-TRIB2. **F, G** Kinetic assay results of the dissociation constant between human TRIB2 and PKM2. **H** Diagram showing the constructs of GST-TRIB2 and its deletion mutants. **I** Co-IP analysis of the mutant domains of TRIB2 involved in the interaction with PKM2. GST-TRIB2 and mutation domains were induced by IPTG and stably expressed in vitro (lower), and co-precipitation experiments incubated with lysates of A549 cells. **J** Diagram showing constructs of GST-PKM2 and its deletion mutants. GST-PKM2 was induced by IPTG and co-precipitation experiments incubated with lysates of A549 cells. **K** Co-IP with TRIB2 antibodies showing the interaction between TRIB2 and PKM2 mutant domain in A549 cells.

The expression of TRIB2, PKM2, and p-PKM2 was higher in the lung cancer cells than that in the controls (Fig. S1E). GST-TRIB2 was then induced by IPTG and stably expressed in Escherichia coli. Co-IP experiments incubated GST-fusion protein with lysates of A549 cells revealed that anti-GST antibodies precipitated GST-TRIB2, together with PKM2 (Fig. 2A), which was further supported in other cancer cells (Fig. S1F). Co-IP experiments with total lysates of A549 cells showed that anti-TRIB2 antibodies precipitated PKM2 (Fig. 2B), suggesting that TRIB2 could interact with PKM2.

When GST-PKM2 was induced by IPTG and stably expressed in vitro, Co-IP experiments showed that anti-GST antibodies precipitated Flag-TRIB2 (Fig. S1G). When incubated with lysates of A549 cells, anti-GST antibodies also precipitated TRIB2 (Fig. 2C). Co-precipitation experiments with lysates of A549 cells revealed that anti-PKM2 antibodies precipitated GFP-TRIB2 in GFP-TRIB2-overexpressed cells (Fig. S1H) and TRIB2 (Fig. 2D), thus supporting that PKM2 can interact with TRIB2.

Immunofluorescence results revealed the partial co-localization of exogenously expressed GFP-TRIB2 with PKM2 in A549 cells (Fig. 2E), thereby supporting that TRIB2 interacts with PKM2. Kinetic assay results from Octet® System (Pall ForteBio LLC, CA, USA) further revealed that the dissociation constant of TRIB2 and PKM2 protein was at 7.44E-09 M (Fig. 2F, G), suggesting that TRIB2 has a high binding affinity to PKM2 protein.

### Mapping the interaction of TRIB2 domain with PKM2 domain

GST-tagged TRIB2 domain deletion mutants were constructed (Fig. 2H), which was induced by IPTG for stable expression and incubated with the lysates of A549 cells. PKM2 mainly precipitated with GST-TRIB2-Full, GST-TRIB2-A (1-308), and GST-TRIB2-D (308–343, mainly in this region), indicating that TRIB2 might mainly interact with PKM2 via its C-terminal region (238–340) (Fig. 2I).

GST-tagged PKM2 domain deletion mutants were constructed to investigate the binding site of PKM2 with TRIB2 (Fig. 2J). Co-IP experiments supported that TRIB2 could mainly bound with GST-PKM2 (241–408) (Fig. 2K). The protein domain deletion mutants further supported the interaction between TRIB2 and PKM2.

### TRIB2 increases and promotes pSer37-PKM2 entering nucleus to drive gene expression

**Fig. 3:**
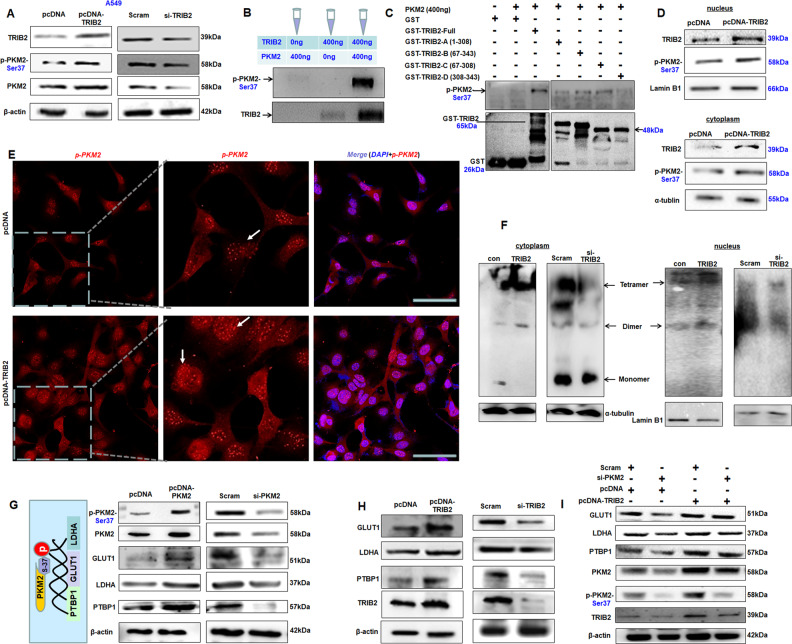
TRIB2 regulates and increases p-PKM2 levels. **A** Immunoblotting analysis of the effect of TRIB2 on pSer37-PKM2. TRIB2 increased pSer37-PKM2 but siRNA reduced pSer37-PKM2 levels. **B** Kinase phosphorylation analysis in vitro. Immunoblotting showed the increased pSer37-PKM2 levels in mixtures added with 400 ng TRIB2 and 400 ng PKM2. **C** GST-TRIB2 mutant analyzing the kinase activity of TRIB2 domain. Compared with other mutants, GST-TRIB2-C (67–308, the central domain) can obviously phosphorylate PKM2 at Ser-37. **D** Effect of TRIB2 on pSer37-PKM2 levels in cytoplasm and nucleus. Overexpression of TRIB2 increased pSer37-PKM2 in the cytoplasm and nucleus. **E** Immunostaining of A549 cells with antibodies against pSer37-PKM2 (red). Scale bar = 100 µm. TRIB2 obviously increased pSer37-PKM2 levels in the nucleus. **F** Tetrameric and dimeric forms of PKM2 in cytoplasm and nucleus. TRIB2 overexpression obviously increased PKM2 dimers in the cytoplasm and nucleus. **G** PKM2 promoted the expression of GLUT1, LDHA, and PTBP1. **H** TRIB2 promoted the expression of GLUT1, LDHA, and PTBP1. **I** Immunoblotting showed that blocking PKM2 suppressed the regulating role of TRIB2 for GLUT1, LDHA, and PTBP1.

The phosphorylation status of PKM-2 at Ser37 helps PKM2 enter the nucleus, and is responsible for the genes that promote the aerobic glycolysis [29]. The levels of pSer37-PKM2 obviously increased in TRIB2-overexpressed A549 cells but decreased in siRNA-treated A549 cultures (Fig. 3A). When TRIB2 and PKM2 proteins were mixed together with ATP in vitro, pSer37-PKM2 levels were obviously increased in TRIB2 and PKM2 protein-mixed tube compared with those in only TRIB2 or PKM2 control group (Fig. 3B), indicating that TRIB2 can directly phosphorylate PKM2 at Ser37. Furthermore, pSer37-PKM2 levels were upregulated in GST-TRIB2-A-, GST-TRIB2-B-, and GST-TRIB2-C-treated reactions but not in GST-TRIB2-D- or GST-treated controls (Fig. 3C). In particular, higher pSer37-PKM2 levels were found in GST-TRIB2-C treated group than in other mutant treatments. These results suggested the central domain (67–308) has kinase activity to phosphorylate PKM2 at Ser37 directly.

Ser37 site phosphorylation is a necessary condition for PKM2 entry in the nucleus to regulate gene expression [29]. The results showed that TRIB2 could increase pSer37-PKM2 levels in the nucleus and cytoplasm (Fig. 3D). Immunofluorescence staining further supported that TRIB2 increased pSer37-PKM2 levels in the nucleus and cytoplasm (Fig. 3E). Signal-regulated kinase 2 (ERK2) can phosphorate PKM2 and increase p-PKM2 levels, which is required for nuclear translocation of PKM2 [12]. Therefore, TRIB2 may be a novel protein that increases pSer37-PKM2 levels and promotes PKM2 dimer entering the nucleus to regulate gene expression [29]. Our results showed PKM2 dimers increased in the cytoplasm and nucleus of TRIB2-overexpressed cells, while siRNA-TRIB2 reduced PKM2 dimers in the cytoplasm and nucleus by native PAGE (Fig. 3F), suggesting that TRIB2 may regulate PKM2 dimer levels by affecting pSer37-PKM2.

The PKM2 in the nucleus further promotes the expression of GLUT1 and LDHA related to aerobic glycolysis [9]. The levels of GLUT1, LDHA, and PTBP1 were increased in PKM2-overexpressed A549 cultures but decreased in PKM2-siRNA-knocked down cells compared with those in controls (Fig. 3G). Moreover, GLUT1, LDHA, and PTBP1 levels were enhanced in TRIB2-overexpressed A549 cells but were reduced in TRIB2-siRNA-knocked down cells compared with controls (Fig. 3H). Blocking PKM2 diminished the roles of TRIB2 in promoting the expression of LDHA, GLUT1, PTBP1, PKM2, and pSer37-PKM2 (Fig. 3I). These findings revealed that TRIB2 could increase pSer37-PKM2, which enters the nucleus to promote the expression of aerobic glycolysis-related genes.

### TRIB2 and PKM2 promotes the aerobic glycolysis in cancer cells

**Fig. 4:**
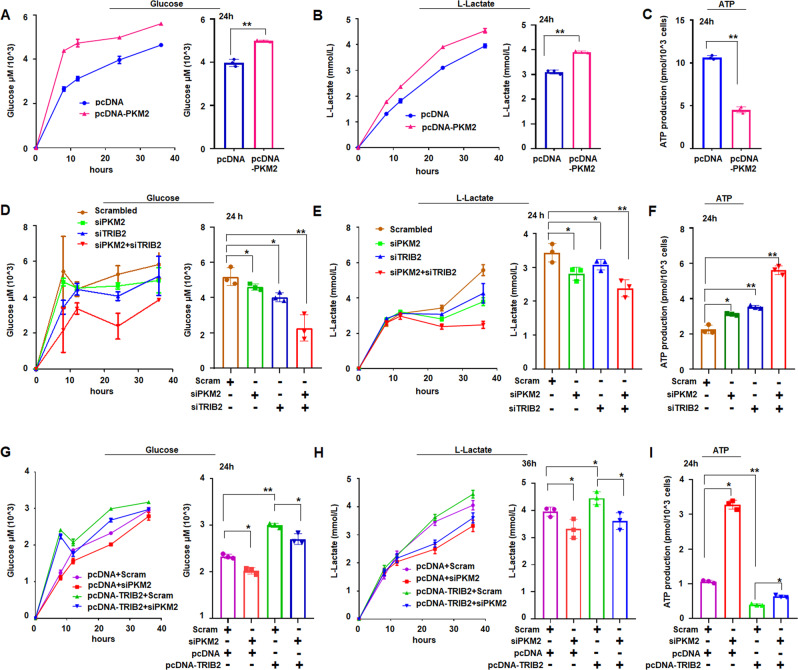
Influence of TRIB2 and PKM2 on the aerobic glycolysis in A549 cells. **A, B** PKM2 increased glucose uptake and lactate production. PKM2 overexpression significantly increased glucose uptake and lactate production at 24 h compared with that in control treatment. Data were expressed as mean ± SD for triplicate experiments. **p < 0.01; Student’s *t* test. **C** PKM2 reduced ATP production at 24 h compared with that in controls. Data were expressed as mean ± SD for triplicate experiments. **p < 0.01; Student’s *t* test. **D, E** siRNA-PKM2 and siRNA-TRIB2 inhibited glucose uptake and lactate production. Downregulation of PKM2, TRIB2, or both, obviously decreased glucose uptake and lactate production at 24 h compared with that in control treatment. Data were expressed as mean ± SD for triplicate experiments. *p < 0.05, **p < 0.01; ANOVA. **F** Downregulation of PKM2, TRIB2, or both, increased ATP production at 24 h compared with that in control treatment. Data were expressed as mean ± SD for triplicate experiments, *p < 0.05, **p < 0.01; ANOVA. **G** Effect of blocking PKM2 on TRIB2 regulating glucose uptake. TRIB2 increased glucose uptake at 24 h compared with that in control treatment, and blocking PKM2 reduced TRIB2-inducing glucose uptake. Data were expressed as mean ± SD for triplicate experiments, *p < 0.05, **p < 0.01; ANOVA. **H** Effect of blocking PKM2 on TRIB2 regulation for lactate levels. TRIB2 increased lactate production at 36 h compared with that in control treatment, and blocking PKM2 reduced the TRIB2-induced lactate levels. Data were expressed as mean ± SD for triplicate experiments, *p < 0.05; ANOVA. **I** TRIB2 downregulated ATP production at 24 h compared with that in controls, and blocking PKM2 increased the TRIB2-induced ATP levels. Data were expressed as mean ± SD for triplicate experiments, *p < 0.05, **p < 0.01; ANOVA.

The aerobic glycolysis is an aberrant metabolism in cancer cells and facilitates cancer cell growth with elevated glucose uptake and lactate production [30]. Our results showed that PKM2 overexpression increased the glucose uptake from culture medium and lactate production released from cells, but reduced the ATP content produced in cells (Fig. 4A–C) in PKM2-treated A549 cells compared with those in controls. When PKM2 was knocked down in A549 cells by siRNA, glucose uptake from culture medium and lactate production from cells were decreased (Fig. 4D, E), but ATP content was relatively increased (Fig. 4F) compared with those in controls. Moreover, knocking down PKM2 can suppress the aerobic glycolysis in H1975 cells (Fig. S2A–C). These results may be related to PKM2 mainly promoting aerobic glycolysis in tumor cells. When TRIB2 was knocked down in A549 cells by siRNA, glucose uptake from culture medium and lactate production released from cells decreased obviously, but ATP content in cells was increased (Fig. 4D–F) compared with those in control treatment. In siRNA-PKM2- and siRNA-TRIB2-treated cells, glucose uptake and lactate production were further decreased, but ATP content was increased significantly (Fig. 4D–F). In A549 cells, TRIB2 overexpression increased glucose uptake and lactate production but reduced ATP levels (Fig. 4G–I) compared with those in controls. Furthermore, the aerobic glycolysis in H1975 cells was suppressed by TRIB2 downregulation but promoted by TRIB2 overexpression (Fig. S2D–F). These results indicated that PKM2 and TRIB2 would promote aerobic glycolysis in lung cancer cells.

Moreover, siRNA-PKM2 treatment decreased glucose uptake and lactate production but increased ATP content in TRIB2-overexpressed A549 or H1975 cells (Figs. 4G–I; S2D–F), indicating that blocking PKM2 can reverse the roles of TRIB2 in inducing aerobic glycolysis. These results supported that TRIB2 could regulate the aerobic glycolysis through PKM2 because blocking PKM2 attenuates the roles of TRIB2 in promoting the expression of GLUT1, LDHA, and PTBP1.

### PKM2 and TRIB2 promote lung cancer cell growth and migration

**Fig. 5:**
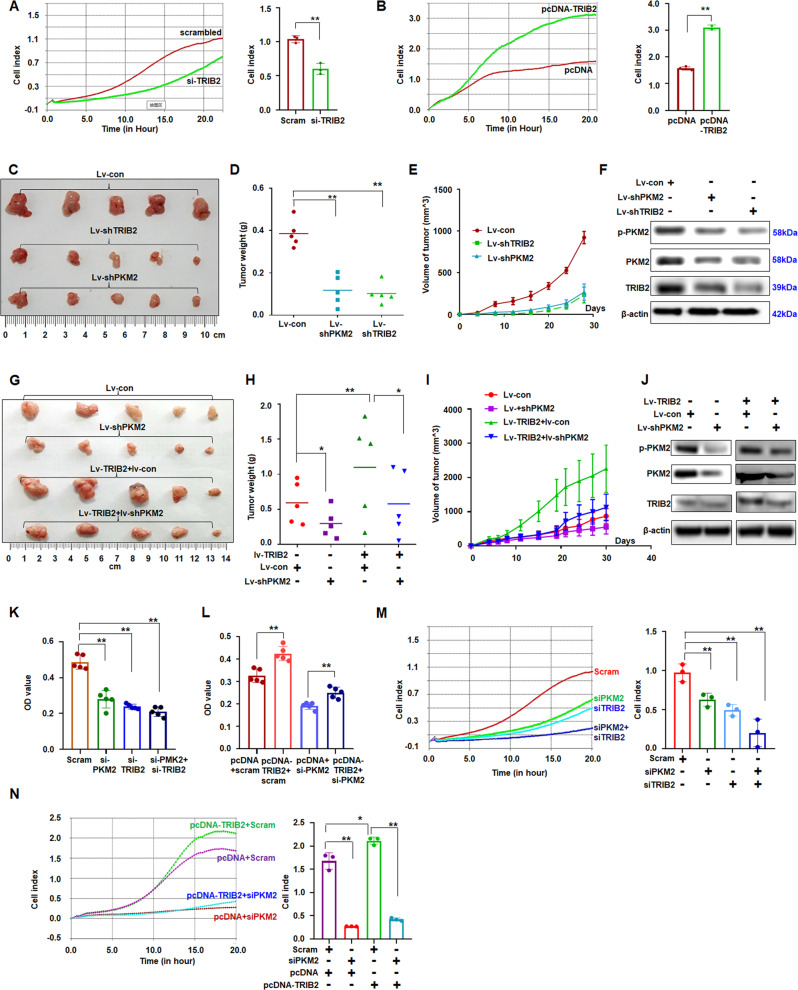
Effect of blocking PKM2 on oncogenic role of TRIB2 in cell growth. **A**, **B** RTCA station analysis of knocking down or overexpression of TRIB2 on regulating A549 cell migration. Data were expressed as mean ± SD for triplicate experiments, **p < 0.01; Student’s *t* test. **C**–**F** Analysis of xenografts of stable A549 cells expressed of lentivirus si-TRIB2, si-PKM2, or controls (*n* = 5). Quantitative data of tumor weight (**D**) and detected tumor volume change (**E**) of xenografts were analyzed. Protein expression was analyzed by immunoblotting (**F**). Data were expressed as median (interquartile range), **p < 0.01; Kruskal–Wallis *H* test. **G**–**I** Analysis of xenograft tumors of lv-TRIB2 with lv-si-PKM2 or control treatment (*n* = 5). Quantitative data of tumor weight (**H**) and detected tumor volume change (**I**) of xenografts were analyzed. Protein expression was analyzed by immunoblotting (**J**). Data were expressed as median (interquartile range), *p < 0.05, **p < 0.01; Kruskal–Wallis *H* test. **K** PKM2 and TRIB2 downregulation obviously inhibited A549 cell proliferation. Data are expressed as the mean ± SD of triplicate experiments, **p < 0.01; ANOVA. **L** Blocking PKM2 attenuated TRIB2-promoting A549 cell proliferation compared with that in control treatment. Data are expressed as the mean ± SD of triplicate experiments, *p < 0.01; ANOVA. **M** RTCA station analysis of siPKM2 and siTRIB2 on regulating cell migration. Migrative cells were counted on the left. Data were expressed as mean ± SD for triplicate experiments, **p < 0.01; ANOVA. **N** RTCA station analysis showed that si-PKM2 blocked the TRIB2-promoting cell migration. Data were expressed as mean ± SD for triplicate experiments, *p < 0.05; **p < 0.01; ANOVA.

TRIB2 with PKM2 promotes the Aerobic glycolysis, generating the energy required to support rapid cancer cell proliferation [5]. The results showed that PKM2 or TRIB2 suppression significantly inhibited lung cancer cell proliferation (Fig. S3A, B) and migration compared with those in controls (Figs. 5A; S3C). Either PKM2 or TRIB2 overexpression increased cancer cell proliferation (Fig. S3D, E) and migration (Figs. 5B; S3F).

The dimeric PKM2 can enter the nucleus to activate HIF-1α-related genes [31, 32]. Here, the protein levels of HIF-1α-related cell proliferation genes (Cyclin D1, c-Myc, and OCT4) were increased in PKM2- or TRIB2-overexpressed cells, but decreased in siRNA-PKM2- or siRNA-TRIB2-treated cells compared with those in controls (Fig. S3G).

The above results showed that PKM2 or TRIB2 can promote lung cancer cell proliferation in vitro. To investigate the roles of PKM2 and TRIB2 in regulating lung cancer cell growth in vivo, lentiviral vectors were then constructed as a previous report [28] to stably express PKM2, shPKM2, TRIB2, shTRIB2, or controls to further investigate the effect of PKM2 or TRIB2 on cancer cell proliferation in xenografts. PKM2 overexpression obviously promoted, whereas PKM2 downregulation significantly inhibited cancer cell proliferation in vivo compared with that in the control treatment (Fig. 5C–F; S3H–J). The levels of p-PKM2 and PKM2 were increased in PKM2-overexpressed xenografts but decreased in lv-shPKM2-treated xenografts (Figs. 5F; S3K). TRIB2 overexpression obviously promoted cancer cell proliferation and increased tumor weight in vivo compared with those in lv-con treatment (Fig. 5G–J). The suppression of TRIB2 with shTRIB2 significantly inhibited cancer cell proliferation in vivo compared with that in lv-con treatment (Fig. 5C–E). The levels of TRIB2, p-PKM2, and PKM2 decreased in lv-shTRIB2-treated xenografts (Fig. 5F).

### Blocking PKM2 attenuates the role of TRIB2 in cell proliferation and migration

Knocking down PKM2 would further strengthen the cell-suppressing function of si-TRIB2. The role of promoting cell proliferation in TRIB2-overexpressed cultures was obviously blocked after si-PKM2 treatment compared with that in the control treatment (Fig. 5K, L). Moreover, knocking down PKM2 further enhanced the suppressive roles of si-TRIB2 in cell migration (Fig. 5M). The promoting role of cell migration in TRIB2-overexpressed cultures was substantially blocked following additional si-PKM2 treatment (Fig. 5N).

Compared with that in controls, TRIB2 overexpression would promote lung cancer cell proliferation, whereas blocking PKM2 attenuated the tumorigenic role of TRIB2 in vivo. The weights and volumes were smaller in lv-TRIB2 + lv-siPKM2-treated xenografts than in tumors treated with lv-TRIB2 + control (Fig. 5G–I). Levels of p-PKM2, PKM2, and TRIB2 decreased in lv-TRIB2 + lv-siPKM2-treated xenografts compared with those in the control treatment (Fig. 5J). These results indicated that blocking PKM2 could attenuate the oncogenic role of TRIB2 in cell proliferation.

### TRIB2 deletion weakens the aerobic glycolysis in TRIB2^−/−^ mice

**Fig. 6:**
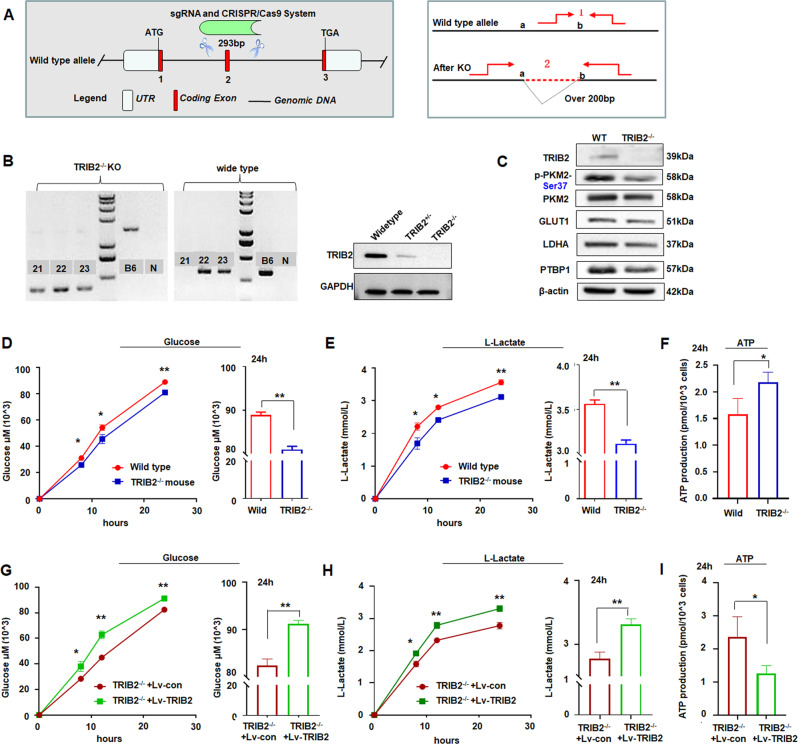
Influence TRIB2 on the Aerobic Glycolysis in TRIB2^−/−^ cells. **A** Sketch map of knockout TRIB2 model by CRISPR/cas9 technology. **B** PCR and immunoblotting results of wild type and model knockout mice. 21–23, TRIB2^−/−^ mice; B6, wide type mice; N, negative control. Immunoblotting results showed that TRIB2^−/−^ mice did not express TRIB2 protein. **C** Immunoblotting analysis revealed that the levels of pSer37-PKM2, PKM2, LDHA, GLUT1, and PTBP1 decreased in TRIB2^−/−^ mice. **D** Glucose uptake decreased in bone marrow cells of TRIB2^−/−^ mice compared with those in wide type. **E** The levels of lactate production were reduced in bone marrow cells of TRIB2^−/−^ mice compared with those in wide type. **F** ATP production. ATP content increased in fibroblasts of TRIB2^−/−^ mice compared with that in wide type. **G** Glucose uptake. TRIB2 expression rescued glucose uptake in fibroblasts of TRIB2^−/−^ mice compared with control. **H** Lactate production. TRIB2 expression increased lactate production in fibroblasts of TRIB2^−/−^ mice compared with control. **I** ATP production. TRIB2 expression decreased ATP content in fibroblasts of TRIB2^−/−^ mice compared with control. Data were expressed as mean ± SD for triplicate experiments. **p < 0.01, *p < 0.05; Student’s *t* test.

Trib2-reconstituted mice uniformly developed fatal transplantable acute myelogenous leukemia [15]. Here, TRIB2^−/−^ mice were prepared to further study the influence of TRIB2 on the aerobic glycolysis (Fig. 6A, B). The levels of pSer37-PKM2, PKM2, and the aerobic glycolysis-related genes were reduced in TRIB2^−/−^ mice (Fig. 6C). The levels of glucose uptake and lactate production were decreased, while ATP content was increased in the cultured bone marrow cells of TRIB2^−/−^ mice compared with those in the wild type (Fig. 6D–F). When TRIB2^−/−^ bone marrow cells were re-expressed with TRIB2, the levels of glucose uptake from culture medium and lactate production from cells were elevated, whereas the ATP content in cells was reduced compared with those in untreated TRIB2^−/−^ cells (Fig. 6G–I), further supporting that TRIB2 re-expression increases glucose uptake and lactate production but decreases ATP content.

## Discussion

**Fig. 7:**
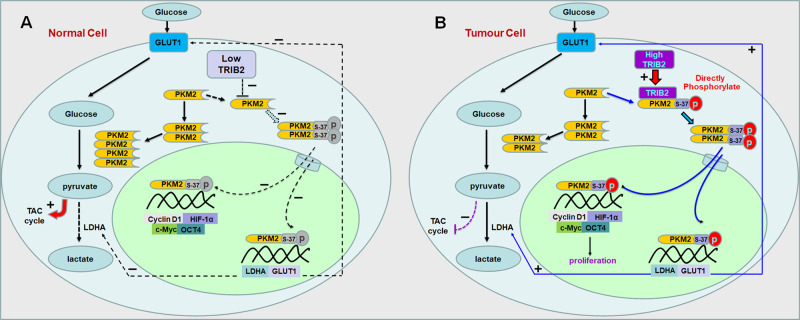
Proposed model by which TRIB2 regulates the aerobic glycolysis and cell proliferation by interacting with PKM2. **A** In normal cells, the levels of TRIB2 and pSer37-PKM2 are relatively low. PKM2 effectively forms tetramers in the cytoplasm and performs high catalytic activity with its substrate PEP. PKM2 catalyzes the terminal step of glycolysis and transfers PEP to pyruvate. Pyruvate is further oxidized via the tricarboxylic acid (TCA) cycle to produce ATP. The low levels of pSer37-PKM2 cannot effectively enter nucleus to drive the aerobic glycolysis-related genes (LDHA, GLUT1, and PTBP1), HIF-1α, Cyclin D, and c-Myc. Therefore, the low levels of TRIB2 inhibit the aerobic glycolysis. **B** In cancer cells, TRIB2 has a relatively high level, interacts with PKM2, and participates in phosphorylating PKM2 at Ser 37. PKM2 effectively forms dimers in the cytoplasm. The dimer structure of PKM2 is regulated by tumor environmental factors, oncogenes, and tumor suppressor genes. The increased dimers indicate that tumor cells accumulate mainly by glycolysis metabolites at this time. The dimeric PKM2 can enter the nucleus to activate the aerobic glycolysis-related genes, HIF-1α, cyclin D, and c-Myc. Therefore, high levels of TRIB2 promote the aerobic glycolysis in cancer cells.

With its pseudo serine/threonine kinase domain, TRIB2 functions as a scaffold or adaptor in the signaling pathways of physiological and pathological processes [33]. Whether TRIB2 participates the regulation of kinase activities in cell metabolism remains unclear. In this study, TRIB2 is found to interact with PKM2 and may exhibit kinase activity to directly phosphorylate PKM2 at serine 37 in cancer cells. pSer37-PKM2 then forms dimers, which would be transferred into nucleus to promote the expression of aerobic glycolysis-related LDHA and GLUT1, HIF-1α-related Cyclin D1, c-Myc, and OCT4 genes. As a result, LDHA and GLUT1 promote the aerobic glycolysis to provide energy for TRIB2-treated lung cancer cells. Moreover, the levels of HIF-1α, Cyclin D1, c-Myc, and OCT4 were elevated in TRIB2-overexpressed cancer cells, which further promote cancer cell proliferation. In contrast, to those in normal cells, a low TRIB2 level cannot elevate pSer37-PKM2 and dimers, and PKM2 tetramers promote pyruvate levels to participate in TAC cycle (Fig. 7).

TRIB2 participates in tumorigenesis. TRIB2 exogenous expression can induce AML in mice, which is related to C/EBPα degradation [34]. TRIB2 is highly expressed in T-ALL [35], and identified as a neurogenic locus Notch homolog protein homologue 1 target [36]. High TRIB2 levels are related to F-box/WD repeat-containing protein 7 mutations in pediatric cases [32]. Additionally, we previously demonstrated that TRIB2 has an oncogenic role in lung adenocarcinoma, and miR-206 can modify TRIB2 promoter activity through p-Smad3 [37]. Here, we further found that a high TRIB2 level is associated with the poor survival of patients with lung cancer and promotes lung cancer proliferation by regulating aerobic glycolysiss via PKM2-related factors.

TRIB2 has an N-terminal domain, a conserved pseudokinase domain, and a C-terminal E3 ligase-binding domain [38], through which TRIB2 regulates cell functions by interacting with various signaling molecules via their different domains [39, 40]. Here, we found that the C-terminal region (238–340) of TRIB2 might mainly interact with PKM2 protein, which is related to regulating the aerobic glycolysis of cancer cells. GST-tagged PKM2 domain deletion mutants also indicated that GST-PKM2 (241–408) would bind with TRIB2.

The C- terminal DQxVPx motif of TRIB2 can bind the E3 ligase COP1 [38]. TRIB2 and TRIB3 have low vestigial ATP affinity and phosphotransferase capacity in vitro [39, 41]. The ability of TRIB2 on involving the phosphorylation of protein substrates in vitro or in vivo is poorly understood. In this study, TRIB2 has elevated the p-PKM2 levels in cancer cells and reduced them in TRIB2^−/−^ mutant mice. Our results demonstrate that TRIB2 might be not a “pseudokinase”, which can promote the phosphorylation of PKM2 directly through its central serine/threonine kinase-like domain.

Aerobic glycolysis increases glucose uptake and produces copious amount of pyruvate lactate via enhanced glycolysis without any regard for oxygen concentration in cancer cells [42]. PKM2 is involved in mediating the aerobic glycolysis in many types of cancer [43]. The phosphorylation status of PKM-2 at Ser37 is translocated into the nucleus, which is necessary for the expression of aerobic glycolysis-related genes (GLUT1 and LDHA) and cell proliferation genes (Cyclin D1 and c-Myc) [44]. Our results supported that high PKM2 levels were related to the poor survival overall of patients with lung cancer. The overexpressed PKM2 and TRIB2 increased the expression of GLUT1, LDHA, and PTBP1 to promote the aerobic glycolysis in lung cancer cells.

PKM2 actually exists as inactive monomer, less active dimer, and active tetramer. Under normal conditions, the tetrameric form has high glycolytic activity [45]. The low catalytic activity of PKM2 dimer produces a build-up of glycolytic intermediates to sustain tumor cell proliferation [46]. Here, our results showed that TRIB2 may have kinase phosphorylation activity to directly increase p-PKM2 levels, PKM2 dimers, and the expression of aerobic glycolysis-related genes. Although this study reveals the phosphorylated kinase activity of TRIB2 and PKM2 as substrate, the other substrates are unclear. The detailed regulating mechanism underlying TRIB2 kinase activity must be further investigated.

In summary, we found that TRIB2 may have phosphorylated kinase activity to directly phosphorylate PKM2 at serine 37 in cancer cells. The elevated pSer37-PKM2 subsequently would promote the dimers to enter into the nucleus and increases the expression of LDHA, GLUT1, and PTBP1. Aerobic glycolysis is then elevated in cancer cells. This study reveals the new kinase activity of TRIB2 and its mechanism in cancer metabolism by regulating PKM2. New targets for cancer therapy by controlling cancer metabolism-related genes are also provided.

## Supplementary information

supplemental figures

supplemental table 1

author contributions

animal Ethics

Medical Ethics

## Data Availability

The datasets used and/or analyzed during the current study are available from the corresponding author on reasonable request.
