# Supplementary material for: Oncogenic TRIB2 interacts with and regulates PKM2 to promote aerobic glycolysis and lung cancer cell procession
Source: Cell Death Discov. 2022 Jul 5;8:306. doi: 10.1038/s41420-022-01095-1 (PMC9256704; doi:10.1038/s41420-022-01095-1)
Supplement: Supplementary file 1 — supplemental figures [file 41420_2022_1095_MOESM1_ESM.pdf]

Oncogenic TRIB2 interacts with and regulates PKM2 to promote aerobic glycolysis and lung cancer cell procession

Supplemental figures and figure legends

Fig. S1

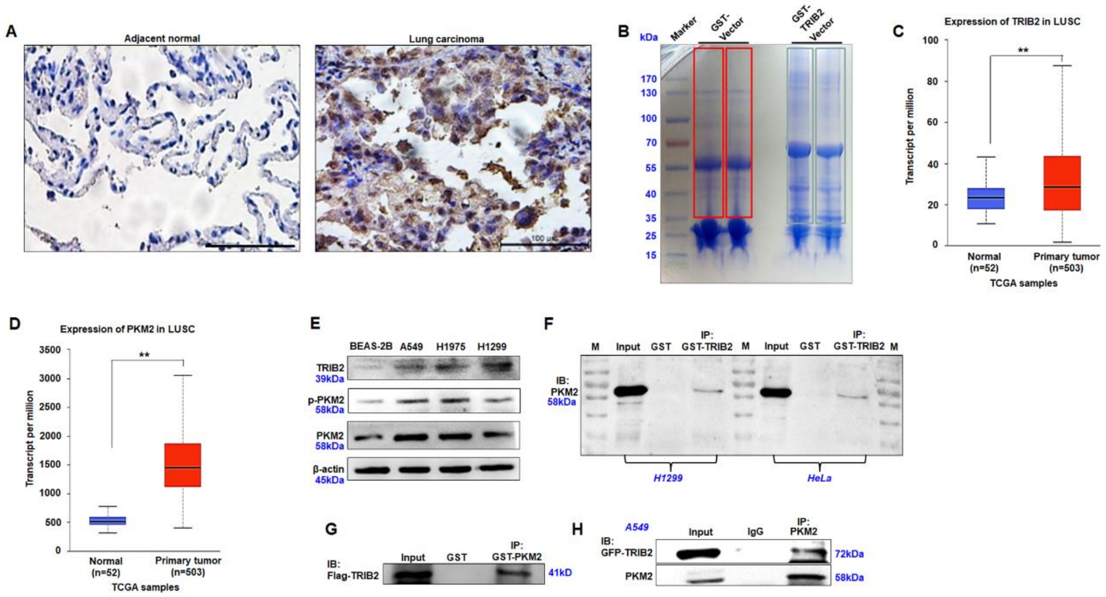

Fig. S1 Analyzing the interaction between TRIB2 and PKM2

A Immunohistochemistry showed TRIB2 expression was high in lung anticarcinoma versus adjacent normal lung tissue, bar = 100 µm.

B SDS-PAGE gel showing proteins co-IP by anti-GST from incubated with the lysis of lung cancer tissues. The gel was stained by Coomassie brilliant blue and differential bands were subjected for mass spectrometry.

C,D Data from TCGA database showed that the expression of TRIB2 and PKM2 was higher in lung carcinoma cell tissues (n=503) compared with those in control

tissures (n=52), and data were shown as median (interquartile range), $^{**}p < 0.01$; Mann–Whitney U test.

E Pearson′s correlation analysis of TRIB2 expression and PKM2 expression (n=501, $^{**}p = 0.0001$).

F Immunoblotting analysis for TRIB2 and PKM2 in BEAS-2B, A549, H1975, and H1299 cells.

G GST-TRIB2 was induced by IPTG and stably expressed *in vitro*, and Co-precipitation experiments incubated with lysates of H1299 and HeLa cells.

H GST-PKM2 expression was induced by IPTG *in vitro*, and Co-precipitation experiments incubated with lysates of Flag-TRIB2-expressed A549 cells.

I Co-IP with PKM2 antibodies in GFP-TRIB2-expressed A549 cells.

Fig. S2

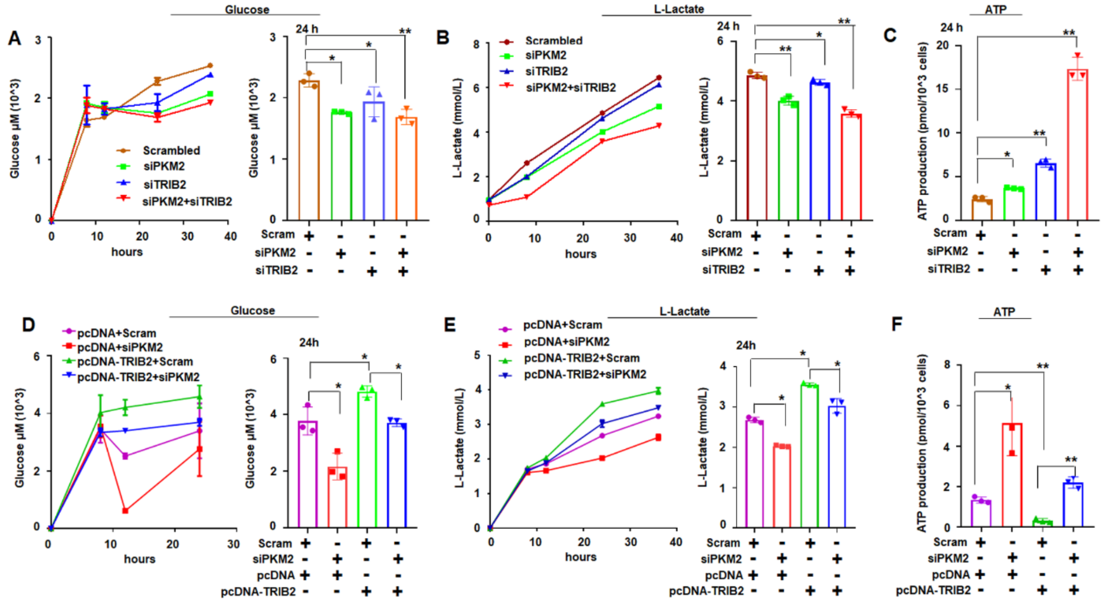

Fig. S2 Effect of TRIB2 and PKM2 on aerobic glycolysis in H1975 cells

A Down-regulation of PKM2, TRIB2, or both, obviously reduced glucose uptake at 24 h in H1975 cells. Data were expressed as mean ± SD for triplicate experiments. $*p < 0.05$, $**p < 0.01$; ANOVA.

B Down-regulation of PKM2, TRIB2, or both, significantly decreased the lactate production at 24 h. Data were expressed as mean ± SD for triplicate experiments. $*p < 0.05$, $**p < 0.01$; ANOVA.

C Down-regulation of PKM2, TRIB2, or both, increased ATP production at 24 h. Data were expressed as mean ± SD for triplicate experiments. $*p < 0.05$, $**p < 0.01$; ANOVA.

D TRIB2 increased glucose uptake at 24 h, and blocking PKM2 reduced TRIB2-induced glucose uptake. Data were expressed as mean ± SD for triplicate experiments. $*p < 0.05$, ANOVA.

E TRIB2 obviously increased lactate production at 24 h, and blocking PKM2 would

reverse TRIB2-induced lactate levels. Data were expressed as mean ± SD for triplicate experiments. *$p < 0.05$; ANOVA.

F TRIB2 downregulated ATP production at 24 h, and blocking PKM2 increased TRIB2-induced ATP levels. Data were expressed as mean ± SD for triplicate experiments. *$p < 0.05$, **$p < 0.01$; ANOVA.

Fig. S3

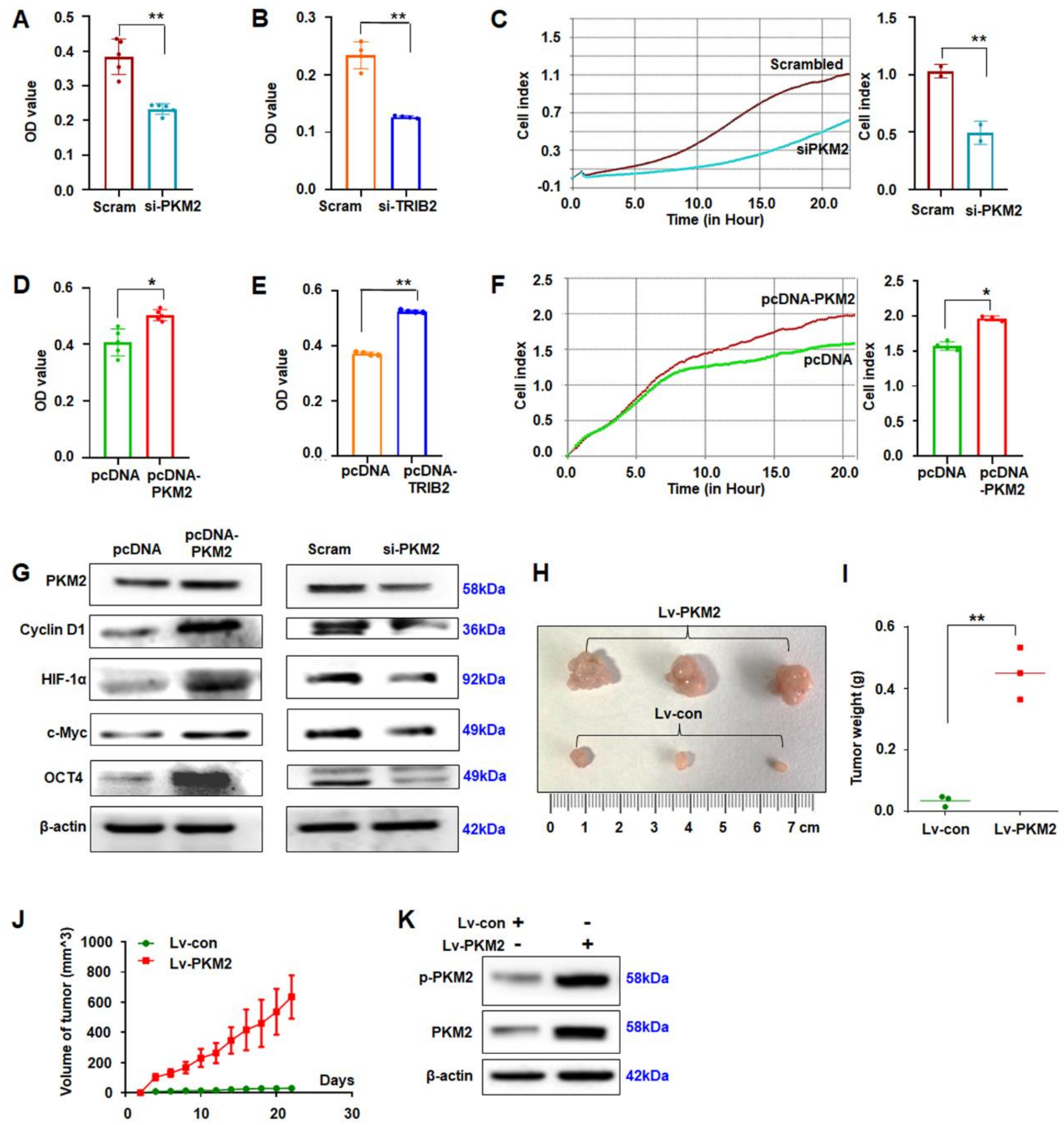

Fig. S3 PKM2 regulating A549 cell growth and migration

A MTT assay. si-PKM2 obviously inhibited A549 cell proliferation compared with scrambled that in control. Data were expressed as mean ± SD for triplicate experiments, $**p < 0.01$; Student's *t*-test.

B MTT assay showed that si-TRIB2 inhibited A549 cell proliferation compared with that in control. Data are expressed as the mean ± SD of triplicate experiments, $**p$

< 0.01; Student's *t*-test.

C RTCA station analysis of migration for A549. Migrative cells were counted on the left. Data were expressed as mean ± SD for triplicate experiments, $**p < 0.01$; Student's *t*-test.

D MTT assay of PKM2 overexpression promoted A549 cell proliferation compared with that in scrambled control. Data were expressed as mean ± SD for triplicate experiments, $*p < 0.05$; Student's *t*-test.

E MTT assay revealed that TRIB2 overexpression increased A549 cell proliferation compared with that in scrambled control. Data are expressed as the mean ± SD of triplicate experiments, $*p < 0.01$; Student's *t*-test.

F RTCA station analysis of PKM2 overexpression promoting cell migration. Migrative cells were counted on the left. Data were expressed as mean ± SD for triplicate experiments, $*p < 0.05$; Student's *t*-test.

G Immunoblotting analysis of the expression of HIF-1α, c-Myc, and OCT4 in PKM2-overexpressed or siRNA-downregulated cells.

H-K Analysis of xenograft tumors of lv-PKM2 and control-treated A549 cells *in vivo* (n=3). Quantitative data of tumor weight (**I**) and dynamically detected volume change (**J**) of xenografts were analyzed. Protein expression was estimated by immunoblotting (**K**). Data were expressed as median (interquartile range), $** p < 0.01$; Mann–Whitney U.
