# Supplementary material for: Oncogenic TRIB2 interacts with and regulates PKM2 to promote aerobic glycolysis and lung cancer cell procession
Source: Cell Death Discov. 2022 Jul 5;8:306. doi: 10.1038/s41420-022-01095-1 (PMC9256704; doi:10.1038/s41420-022-01095-1)
Supplement: Supplementary file 2 — supplemental table 1 [file 41420_2022_1095_MOESM2_ESM.pdf]

Oncogenic TRIB2 interacts with and regulates PKM2 to promote aerobic glycolysis and lung cancer cell procession

Table S1. The biochemical metabolism related proteins interacting with TRIB2

No	Description	Score
1	Pyruvate kinase PKM OS=Homo sapiens GN=PKM2	262.89
2	Glyceraldehyde-3-phosphate dehydrogenase OS=Homo sapiens GN=GAPDH	207.13
3	Carbonyl reductase [NADPH] 1 OS=Homo sapiens GN=CBR1	202.17
4	ATP synthase subunit alpha, mitochondrial OS=Homo sapiens GN=ATP5A1	170.29
5	POTE ankyrin domain family member E OS=Homo sapiens GN=POTEE PE=1 SV=3 - [POTEE_HUMAN]	156.87
6	Four and a half LIM domains protein 1 (Fragment) OS=Homo sapiens GN=FHL1 PE=1 SV=1 - [Q5JXI8_HUMAN]	156.60
7	Selenium-binding protein 1 OS=Homo sapiens GN=SELENBP1 PE=1 SV=2 - [SBP1_HUMAN]	154.64
8	Ig gamma-3 chain C region (Fragment) OS=Homo sapiens GN=IGHG3 PE=1 SV=1 - [A0A075B6N8_HUMAN]	154.64
9	POTE ankyrin domain family member F OS=Homo sapiens GN=POTEF PE=1 SV=2 - [POTEF_HUMAN]	150.54
10	Keratin, type I cytoskeletal 17 OS=Homo sapiens GN=KRT17 PE=1 SV=2 - [K1C17_HUMAN]	147.18
11	Alpha-2-macroglobulin OS=Homo sapiens GN=A2M PE=1 SV=3 - [A2MG_HUMAN]	146.77
12	EH domain-containing protein 2 OS=Homo sapiens GN=EHD2 PE=1 SV=2 - [EHD2_HUMAN]	146.39
13	Ig gamma-4 chain C region OS=Homo sapiens GN=IGHG4 PE=1 SV=1 - [IGHG4_HUMAN]	138.08
14	Chloride intracellular channel protein 5 OS=Homo sapiens GN=CLIC5 PE=1 SV=3 - [CLIC5_HUMAN]	95.13
15	Spectrin alpha chain, non-erythrocytic 1 OS=Homo sapiens GN=SPTAN1 PE=1 SV=3 - [SPTN1_HUMAN]	90.48
16	Ig lambda-1 chain C regions (Fragment) OS=Homo sapiens GN=IGLC1 PE=4 SV=1 - [A0A075B6K8_HUMAN]	81.56
17	Ig lambda-2 chain C regions (Fragment) OS=Homo sapiens GN=IGLC2 PE=4 SV=1 - [A0A075B6K9_HUMAN]	80.02
18	Very long-chain specific acyl-CoA dehydrogenase, mitochondrial OS=Homo sapiens GN=ACADVL PE=1 SV=1 - [ACADV_HUMAN]	78.12
19	Desmoplakin OS=Homo sapiens GN=DSP PE=1 SV=3 - [DESP_HUMAN]	77.30
20	Glutathione S-transferase Mu 1 OS=Homo sapiens GN=GSTM1 PE=1 SV=3 - [GSTM1_HUMAN]	75.77
21	Alpha-1-antitrypsin OS=Homo sapiens GN=SERPINA1 PE=1 SV=3 - [A1AT_HUMAN]	74.90
22	Receptor of activated protein C kinase 1 OS=Homo sapiens GN=RACK1 PE=1 SV=3 - [RACK1_HUMAN]	69.75
23	Unconventional myosin-Ic OS=Homo sapiens GN=MYO1C PE=1 SV=1 - [F5H6E2_HUMAN]	69.35
24	Ig alpha-1 chain C region OS=Homo sapiens GN=IGHA1 PE=1 SV=2 - [IGHA1_HUMAN]	68.84
25	Amine oxidase [flavin-containing] A OS=Homo sapiens GN=MAOA PE=1 SV=1 - [AOFA_HUMAN]	65.72
26	Beta-actin-like protein 2 OS=Homo sapiens GN=ACTBL2 PE=1 SV=2 -	60.79

	[ACTBL_HUMAN]	
27	Junction plakoglobin OS=Homo sapiens GN=JUP PE=1 SV=3 - [PLAK_HUMAN]	59.82
28	Tryptophan--tRNA ligase, cytoplasmic OS=Homo sapiens GN=WARS PE=1 SV=2 - [SYWC_HUMAN]	59.54
29	Tensin-1 OS=Homo sapiens GN=TNS1 PE=1 SV=1 - [E9PGF5_HUMAN]	59.16
30	Histidine-rich glycoprotein OS=Homo sapiens GN=HRG PE=1 SV=1 - [HRG_HUMAN]	57.92
31	Collagen alpha-3(VI) chain OS=Homo sapiens GN=COL6A3 PE=1 SV=2 - [E7ENL6_HUMAN]	57.61
32	Fibroleukin OS=Homo sapiens GN=FGL2 PE=1 SV=1 - [FGL2_HUMAN]	57.41
33	Erythrocyte membrane protein band 4.2 OS=Homo sapiens GN=EPB42 PE=1 SV=3 - [EPB42_HUMAN]	55.95
34	Band 3 anion transport protein OS=Homo sapiens GN=SLC4A1 PE=1 SV=3 - [B3AT_HUMAN]	54.74
35	Talin-1 OS=Homo sapiens GN=TLN1 PE=1 SV=3 - [TLN1_HUMAN]	54.22
36	Ig kappa chain C region OS=Homo sapiens GN=IGKC PE=1 SV=1 - [IGKC_HUMAN]	53.49
37	Ig mu chain C region OS=Homo sapiens GN=IGHM PE=1 SV=1 - [A0A087X2C0_HUMAN]	53.01
38	Glutathione reductase, mitochondrial OS=Homo sapiens GN=GSR PE=1 SV=2 - [GSHR_HUMAN]	52.83
39	60 kDa heat shock protein, mitochondrial OS=Homo sapiens GN=HSPD1 PE=1 SV=2 - [CH60_HUMAN]	52.36
40	40S ribosomal protein S3 OS=Homo sapiens GN=RPS3 PE=1 SV=2 - [RS3_HUMAN]	51.48
41	Glutathione S-transferase Mu 5 OS=Homo sapiens GN=GSTM5 PE=1 SV=3 - [GSTM5_HUMAN]	51.02
42	Biglycan OS=Homo sapiens GN=BGN PE=1 SV=2 - [PGS1_HUMAN]	50.75
43	40S ribosomal protein S4, X isoform OS=Homo sapiens GN=RPS4X PE=1 SV=2 - [RS4X_HUMAN]	50.41
44	Prelamin-A/C OS=Homo sapiens GN=LMNA PE=1 SV=1 - [LMNA_HUMAN]	49.74
45	Plastin-3 OS=Homo sapiens GN=PLS3 PE=1 SV=4 - [PLST_HUMAN]	49.51
46	Glucose-6-phosphate 1-dehydrogenase OS=Homo sapiens GN=G6PD PE=1 SV=4 - [G6PD_HUMAN]	49.29
47	Keratin, type II cytoskeletal 8 OS=Homo sapiens GN=KRT8 PE=1 SV=7 - [K2C8_HUMAN]	48.10
48	F-actin-capping protein subunit alpha-2 OS=Homo sapiens GN=CAPZA2 PE=1 SV=3 - [CAZA2_HUMAN]	47.51
49	F-actin-capping protein subunit alpha-1 OS=Homo sapiens GN=CAPZA1 PE=1 SV=3 - [CAZA1_HUMAN]	46.90
50	Retinal dehydrogenase 1 OS=Homo sapiens GN=ALDH1A1 PE=1 SV=2 - [AL1A1_HUMAN]	46.88
51	Filamin-B OS=Homo sapiens GN=FLNB PE=1 SV=2 - [FLNB_HUMAN]	46.42
52	Spectrin beta chain, non-erythrocytic 1 OS=Homo sapiens GN=SPTBN1 PE=1 SV=2 - [SPTB2_HUMAN]	45.77
53	Hornerin OS=Homo sapiens GN=HRNR PE=1 SV=2 - [HORN_HUMAN]	44.28
54	Epididymis luminal protein 189 OS=Homo sapiens GN=DKFZp686J1372 PE=1 SV=1 - [Q5HYB6_HUMAN]	43.48
55	Myosin-9 OS=Homo sapiens GN=MYH9 PE=1 SV=4 - [MYH9_HUMAN]	42.88
56	3-ketoacyl-CoA thiolase, mitochondrial OS=Homo sapiens GN=ACAA2 PE=1 SV=1 - [A0A0B4J2A4_HUMAN]	42.28

57	Desmoglein-1 OS=Homo sapiens GN=DSG1 PE=1 SV=2 - [DSG1_HUMAN]	41.70
58	Keratin, type II cytoskeletal 7 OS=Homo sapiens GN=KRT7 PE=1 SV=5 - [K2C7_HUMAN]	40.67
59	Peroxiredoxin-2 OS=Homo sapiens GN=PRDX2 PE=1 SV=5 - [PRDX2_HUMAN]	40.40
60	Membrane primary amine oxidase OS=Homo sapiens GN=AOC3 PE=1 SV=3 - [AOC3_HUMAN]	39.36
61	Myoferlin OS=Homo sapiens GN=MYOF PE=1 SV=1 - [MYOF_HUMAN]	38.81
62	Desmin OS=Homo sapiens GN=DES PE=1 SV=3 - [DESM_HUMAN]	38.40
63	Actin-related protein 2/3 complex subunit 1B OS=Homo sapiens GN=ARPC1B PE=1 SV=3 - [ARC1B_HUMAN]	37.79
64	Heat shock protein HSP 90-beta OS=Homo sapiens GN=HSP90AB1 PE=1 SV=4 - [HS90B_HUMAN]	37.76
65	Heterogeneous nuclear ribonucleoprotein K (Fragment) OS=Homo sapiens GN=HNRNPK PE=1 SV=1 - [Q5T6W2_HUMAN]	37.73
67	EH domain-containing protein 1 OS=Homo sapiens GN=EHD1 PE=1 SV=2 - [EHD1_HUMAN]	37.36
68	Elongation factor 2 OS=Homo sapiens GN=EEF2 PE=1 SV=4 - [EF2_HUMAN]	37.29
69	Leukocyte elastase inhibitor OS=Homo sapiens GN=SERPINB1 PE=1 SV=1 - [ILEU_HUMAN]	36.94
70	Probable ATP-dependent RNA helicase DDX6 OS=Homo sapiens GN=DDX6 PE=1 SV=2 - [DDX6_HUMAN]	36.39
71	Clathrin heavy chain 1 OS=Homo sapiens GN=CLTC PE=1 SV=5 - [CLH1_HUMAN]	35.83
72	Alpha-enolase OS=Homo sapiens GN=ENO1 PE=1 SV=2 - [ENOA_HUMAN]	35.63
73	Vitamin D-binding protein OS=Homo sapiens GN=GC PE=1 SV=1 - [VTDB_HUMAN]	35.24
74	DDB1- and CUL4-associated factor 11 OS=Homo sapiens GN=DCAF11 PE=1 SV=1 - [DCA11_HUMAN]	34.76
86	Probable ATP-dependent RNA helicase DDX6 OS=Homo sapiens GN=DDX6	36.39
87	Transketolase OS=Homo sapiens GN=TKT	34.62
88	Succinate dehydrogenase [ubiquinone] flavoprotein subunit, mitochondrial OS=Homo sapiens GN=SDHA	34.51
89	NADH-cytochrome b5 reductase 1 OS=Homo sapiens GN=CYB5R1	29.52
90	Fatty acid synthase OS=Homo sapiens GN=FASN	29.41
91	Cytochrome b-c1 complex subunit 2, mitochondrial OS=Homo sapiens GN=UQCRC2	28.85
92	L-lactate dehydrogenase A chain OS=Homo sapiens GN=LDHA	28.45
93	Filaggrin-2 OS=Homo sapiens GN=FLG2	24.55
94	DNA damage-binding protein 1 OS=Homo sapiens GN=DDB1	24.47
95	Fermitin family homolog 2 OS=Homo sapiens GN=FERMT2	24.2
96	Clusterin OS=Homo sapiens GN=CLU	23.21
97	Plasminogen OS=Homo sapiens GN=PLG	22.94
98	Histone H4 OS=Homo sapiens GN=HIST1H4A PE=1 SV=2 - [H4_HUMAN]	22.62
99	Band 4.1-like protein 2 OS=Homo sapiens GN=EPB41L2 PE=1 SV=1 - [I6L9B1_HUMAN]	22.2
100	Vitronectin OS=Homo sapiens GN=VTN PE=1 SV=1 - [VTNC_HUMAN]	22.04
101	Radixin OS=Homo sapiens GN=RDX PE=1 SV=1 - [RADI_HUMAN]	21.86
102	Decorin OS=Homo sapiens GN=DCN PE=1 SV=1 - [PGS2_HUMAN]	21.86
103	Elongation factor 1-beta OS=Homo sapiens GN=EEF1B2 PE=1 SV=3 - [EF1B_HUMAN]	21.8

104	Histone H2B type 1-J OS=Homo sapiens GN=HIST1H2BJ PE=1 SV=3 - [H2B1J_HUMAN]	21.75
105	Peroxisomal multifunctional enzyme type 2 OS=Homo sapiens GN=HSD17B4 PE=1 SV=3 - [DHB4_HUMAN]	21.59
106	HLA class I histocompatibility antigen, alpha chain E OS=Homo sapiens GN=HLA-E PE=1 SV=3 - [HLAE_HUMAN]	21.57
107	EH domain-containing protein 4 OS=Homo sapiens GN=EHD4 PE=1 SV=1 - [EHD4_HUMAN]	21.55
108	Chloride intracellular channel protein 2 OS=Homo sapiens GN=CLIC2 PE=1 SV=3 - [CLIC2_HUMAN]	21.5
109	Erythrocyte band 7 integral membrane protein OS=Homo sapiens GN=STOM PE=1 SV=3 - [STOM_HUMAN]	21.46
110	Desmocollin-1 OS=Homo sapiens GN=DSC1 PE=1 SV=2 - [DSC1_HUMAN]	20.74
111	Keratin, type II cytoskeletal 80 OS=Homo sapiens GN=KRT80 PE=1 SV=2 - [K2C80_HUMAN]	20.65
112	Collagen alpha-1(VI) chain OS=Homo sapiens GN=COL6A1 PE=1 SV=1 - [A0A087X0S5_HUMAN]	20.54
113	Annexin A3 OS=Homo sapiens GN=ANXA3 PE=1 SV=3 - [ANXA3_HUMAN]	20.33
114	Keratin, type I cuticular Ha5 OS=Homo sapiens GN=KRT35 PE=1 SV=1 - [C4AM86_HUMAN]	20.08
115	Mimecan OS=Homo sapiens GN=OGN PE=1 SV=1 - [MIME_HUMAN]	20
116	Actin-related protein 2/3 complex subunit 2 OS=Homo sapiens GN=ARPC2 PE=1 SV=1 - [ARPC2_HUMAN]	19.98
117	EH domain-containing protein 3 OS=Homo sapiens GN=EHD3 PE=1 SV=2 - [EHD3_HUMAN]	19.87
118	Chloride intracellular channel protein 1 OS=Homo sapiens GN=CLIC1 PE=1 SV=4 - [CLIC1_HUMAN]	19.86
119	WD repeat-containing protein 1 OS=Homo sapiens GN=WDR1 PE=1 SV=4 - [WDR1_HUMAN]	19.34
120	Heterogeneous nuclear ribonucleoprotein A3 OS=Homo sapiens GN=HNRNPA3 PE=1 SV=2 - [ROA3_HUMAN]	19.22
121	Polyubiquitin-C (Fragment) OS=Homo sapiens GN=UBC PE=1 SV=7 - [F5H6Q2_HUMAN]	19.05
122	Cysteine and glycine-rich protein 1 OS=Homo sapiens GN=CSRP1 PE=1 SV=3 - [CSRP1_HUMAN]	18.98
123	Sulfide:quinone oxidoreductase, mitochondrial OS=Homo sapiens GN=SQRDL PE=1 SV=1 - [SQRD_HUMAN]	18.91
124	Keratin, type I cuticular Ha3-I OS=Homo sapiens GN=KRT33A PE=2 SV=2 - [KT33A_HUMAN]	18.66
125	Guanine nucleotide-binding protein G(k) subunit alpha OS=Homo sapiens GN=GNAI3 PE=1 SV=3 - [GNAI3_HUMAN]	18.58
126	14-3-3 protein theta OS=Homo sapiens GN=YWHAQ PE=1 SV=1 - [1433T_HUMAN]	18.47
127	Histone H2A type 1-H OS=Homo sapiens GN=HIST1H2AH PE=1 SV=3 - [H2A1H_HUMAN]	18.28
128	Serpin B9 OS=Homo sapiens GN=SERPINB9 PE=1 SV=1 - [SPB9_HUMAN]	18.19
129	Keratin, type II cuticular Hb2 OS=Homo sapiens GN=KRT82 PE=3 SV=3 - [KRT82_HUMAN]	18.13
130	Cystatin-B OS=Homo sapiens GN=CSTB PE=1 SV=2 - [CYTB_HUMAN]	17.96
131	Protein disulfide-isomerase A6 OS=Homo sapiens GN=PDIA6 PE=1 SV=1 - [PDIA6_HUMAN]	17.93
132	2',3'-cyclic-nucleotide 3'-phosphodiesterase OS=Homo sapiens GN=CNP PE=1 SV=2 - [CN37_HUMAN]	17.83

133	Eukaryotic initiation factor 4A-III OS=Homo sapiens GN=EIF4A3 PE=1 SV=4 - [IF4A3_HUMAN]	17.73
134	Keratin, type I cuticular Ha6 OS=Homo sapiens GN=KRT36 PE=2 SV=1 - [KRT36_HUMAN]	17.69
135	Eukaryotic initiation factor 4A-I (Fragment) OS=Homo sapiens GN=EIF4A1 PE=1 SV=1 - [J3QS69_HUMAN]	17.46
136	Adenosylhomocysteinase OS=Homo sapiens GN=AHCY PE=1 SV=4 - [SAHH_HUMAN]	17.43
137	Alpha-actinin-4 OS=Homo sapiens GN=ACTN4 PE=1 SV=2 - [ACTN4_HUMAN]	17.3
138	Acyl-coenzyme A thioesterase 9, mitochondrial OS=Homo sapiens GN=ACOT9 PE=1 SV=2 - [ACOT9_HUMAN]	17.18
139	Eukaryotic initiation factor 4A-II OS=Homo sapiens GN=EIF4A2 PE=1 SV=1 - [E7EQG2_HUMAN]	17.07
140	Voltage-dependent anion-selective channel protein 2 (Fragment) OS=Homo sapiens GN=VDAC2 PE=1 SV=1 - [A0A0A0MR02_HUMAN]	16.92
141	ARP2 actin-related protein 2 homolog (Yeast), isoform CRA_d OS=Homo sapiens GN=ACTR2 PE=1 SV=2 - [F5H6T1_HUMAN]	16.85
142	Dolichyl-diphosphooligosaccharide--protein glycosyltransferase subunit 1 OS=Homo sapiens GN=RPN1 PE=1 SV=1 - [RPN1_HUMAN]	16.66
143	Alpha-parvin (Fragment) OS=Homo sapiens GN=PARVA PE=1 SV=1 - [E9PS97_HUMAN]	16.26
144	Peroxiredoxin-6 OS=Homo sapiens GN=PRDX6 PE=1 SV=3 - [PRDX6_HUMAN]	16.21
145	40S ribosomal protein S2 OS=Homo sapiens GN=RPS2 PE=1 SV=2 - [RS2_HUMAN]	16.12
146	ADP/ATP translocase 2 OS=Homo sapiens GN=SLC25A5 PE=1 SV=7 - [ADT2_HUMAN]	16.04
147	Alpha-actinin-1 OS=Homo sapiens GN=ACTN1 PE=1 SV=1 - [H9KV75_HUMAN]	16.02
148	Probable ATP-dependent RNA helicase DDX5 OS=Homo sapiens GN=DDX5 PE=1 SV=1 - [J3KTA4_HUMAN]	15.97
149	Hydroxymethylglutaryl-CoA lyase, mitochondrial OS=Homo sapiens GN=HMGCL PE=1 SV=2 - [HMGCL_HUMAN]	15.93
150	L-lactate dehydrogenase B chain OS=Homo sapiens GN=LDHB PE=1 SV=2 - [LDHB_HUMAN]	15.89
151	Guanine nucleotide-binding protein G(i) subunit alpha-1 OS=Homo sapiens GN=GNAI1 PE=1 SV=2 - [GNAI1_HUMAN]	15.74
152	Septin-2 OS=Homo sapiens GN=SEPT2 PE=1 SV=1 - [B5MCX3_HUMAN]	15.73
153	Unconventional myosin-Ib OS=Homo sapiens GN=MYO1B PE=1 SV=1 - [E9PDF6_HUMAN]	15.37
154	Serine/threonine-protein phosphatase 2A 55 kDa regulatory subunit B alpha isoform OS=Homo sapiens GN=PPP2R2A PE=1 SV=1 - [2ABA_HUMAN]	15.36
155	Putative transferase CAF17, mitochondrial OS=Homo sapiens GN=IBA57 PE=1 SV=1 - [CAF17_HUMAN]	15.35
156	rRNA 2'-O-methyltransferase fibrillarin OS=Homo sapiens GN=FBL PE=1 SV=2 - [FBRL_HUMAN]	15.33
157	Polymerase I and transcript release factor OS=Homo sapiens GN=PTRF PE=1 SV=1 - [PTRF_HUMAN]	15.22
158	HLA class II histocompatibility antigen, DR alpha chain OS=Homo sapiens GN=HLA-DRA PE=1 SV=1 - [A0A0G2JH46_HUMAN]	15.01
159	NADH dehydrogenase [ubiquinone] 1 alpha subcomplex subunit 10, mitochondrial OS=Homo sapiens GN=NDUFA10 PE=1 SV=1 - [A0A087WXC5_HUMAN]	14.92
160	Guanine nucleotide-binding protein G(I)/G(S)/G(T) subunit beta-2 (Fragment) OS=Homo sapiens GN=GNB2 PE=1 SV=1 - [C9JXA5_HUMAN]	14.9

161	Fructose-1,6-bisphosphatase 1 OS=Homo sapiens GN=FBP1 PE=1 SV=5 - [F16P1_HUMAN]	14.77
162	Rho GDP-dissociation inhibitor 1 OS=Homo sapiens GN=ARHGDIA PE=1 SV=1 - [J3KRE2_HUMAN]	14.64
163	ADP/ATP translocase 3 OS=Homo sapiens GN=SLC25A6 PE=1 SV=4 - [ADT3_HUMAN]	14.59
164	Ribose-phosphate pyrophosphokinase 1 OS=Homo sapiens GN=PRPS1 PE=1 SV=2 - [PRPS1_HUMAN]	14.56
165	Putative keratin-87 protein OS=Homo sapiens GN=KRT87P PE=5 SV=4 - [KR87P_HUMAN]	14.26
166	Tropomyosin alpha-1 chain OS=Homo sapiens GN=TPM1 PE=1 SV=2 - [F5H7S3_HUMAN]	13.99
167	GTP-binding protein Di-Ras2 OS=Homo sapiens GN=DIRAS2 PE=1 SV=1 - [DIRA2_HUMAN]	13.95
168	Keratin, type II cuticular Hb5 OS=Homo sapiens GN=KRT85 PE=1 SV=1 - [KRT85_HUMAN]	13.82
169	Prenylcysteine oxidase 1 OS=Homo sapiens GN=PCYOX1 PE=1 SV=3 - [PCYOX_HUMAN]	13.8
170	HLA class II histocompatibility antigen, DRB1-4 beta chain OS=Homo sapiens GN=HLA-DRB1 PE=1 SV=1 - [2B14_HUMAN]	13.69
171	Phosphoglycerate kinase 1 OS=Homo sapiens GN=PGK1 PE=1 SV=3 - [PGK1_HUMAN]	13.62
172	Complement C4-A OS=Homo sapiens GN=C4A PE=1 SV=1 - [A0A140TA32_HUMAN]	13.62
173	Heterogeneous nuclear ribonucleoprotein L OS=Homo sapiens GN=HNRNPL PE=1 SV=2 - [HNRPL_HUMAN]	13.54
174	DnaJ homolog subfamily A member 1 OS=Homo sapiens GN=DNAJA1 PE=1 SV=2 - [DNJA1_HUMAN]	13.49
175	Pulmonary surfactant-associated protein A2 OS=Homo sapiens GN=SFTPA2 PE=1 SV=1 - [SFPA2_HUMAN]	13.44
176	Keratin, type I cytoskeletal 18 OS=Homo sapiens GN=KRT18 PE=1 SV=1 - [F8VZY9_HUMAN]	13.22
177	Complement C4-B OS=Homo sapiens GN=C4B_2 PE=1 SV=1 - [A0A0G2JL54_HUMAN]	13.11
178	Galectin-3 OS=Homo sapiens GN=LGALS3 PE=1 SV=5 - [LEG3_HUMAN]	13.11
179	Myosin light chain kinase, smooth muscle OS=Homo sapiens GN=MYLK PE=1 SV=4 - [MYLK_HUMAN]	13.02
180	Ras-related C3 botulinum toxin substrate 2 OS=Homo sapiens GN=RAC2 PE=1 SV=1 - [B1AH77_HUMAN]	13.02
181	Suprabasin OS=Homo sapiens GN=SBSN PE=1 SV=2 - [SBSN_HUMAN]	12.97
182	Collagen alpha-2(VI) chain OS=Homo sapiens GN=COL6A2 PE=1 SV=4 - [CO6A2_HUMAN]	12.96
183	ADP/ATP translocase 1 OS=Homo sapiens GN=SLC25A4 PE=1 SV=4 - [ADT1_HUMAN]	12.95
184	Heat shock 70 kDa protein 12B OS=Homo sapiens GN=HSPA12B PE=1 SV=2 - [Q5JX83_HUMAN]	12.88
185	14-3-3 protein epsilon OS=Homo sapiens GN=YWHAE PE=1 SV=1 - [1433E_HUMAN]	12.79
186	GTP-binding nuclear protein Ran OS=Homo sapiens GN=RAN PE=1 SV=3 - [RAN_HUMAN]	12.66
187	Ribonuclease inhibitor OS=Homo sapiens GN=RNH1 PE=1 SV=2 - [RINI_HUMAN]	12.52
188	Phosphoglucomutase-like protein 5 OS=Homo sapiens GN=PGM5 PE=1 SV=2 -	12.46

	[PGM5_HUMAN]	
189	Polypyrimidine tract binding protein 1, isoform CRA_b OS=Homo sapiens GN=PTBP1 PE=1 SV=4 - [A6NLN1_HUMAN]	12.46
190	Voltage-dependent anion-selective channel protein 3 OS=Homo sapiens GN=VDAC3 PE=1 SV=1 - [VDAC3_HUMAN]	12.25
191	Calcium/calmodulin-dependent protein kinase (CaM kinase) II delta, isoform CRA_e OS=Homo sapiens GN=CAMK2D PE=1 SV=1 - [D6R938_HUMAN]	12.11
192	Serine/threonine-protein phosphatase PP1-beta catalytic subunit OS=Homo sapiens GN=PPP1CB PE=1 SV=3 - [PP1B_HUMAN]	12.05
193	Mitochondrial 2-oxoglutarate/malate carrier protein (Fragment) OS=Homo sapiens GN=SLC25A11 PE=1 SV=1 - [I3L1P8_HUMAN]	11.98
194	EPB41 protein OS=Homo sapiens GN=EPB41 PE=1 SV=2 - [Q4VB86_HUMAN]	11.97
195	UTP--glucose-1-phosphate uridylyltransferase (Fragment) OS=Homo sapiens GN=UGP2 PE=1 SV=7 - [C9JTZ5_HUMAN]	11.8
196	Lysozyme C OS=Homo sapiens GN=LYZ PE=1 SV=1 - [LYSC_HUMAN]	11.78
197	Pyruvate dehydrogenase E1 component subunit alpha, somatic form, mitochondrial OS=Homo sapiens GN=PDHA1 PE=1 SV=3 - [ODPA_HUMAN]	11.75
198	Guanine nucleotide-binding protein subunit alpha-13 OS=Homo sapiens GN=GNA13 PE=1 SV=2 - [GNA13_HUMAN]	11.67
199	Neutrophil defensin 1 OS=Homo sapiens GN=DEFA1 PE=1 SV=1 - [DEF1_HUMAN]	11.61
200	Napsin-A OS=Homo sapiens GN=NAPSA PE=1 SV=1 - [NAPSA_HUMAN]	11.6
201	60S ribosomal protein L18a (Fragment) OS=Homo sapiens GN=RPL18A PE=1 SV=1 - [M0R3D6_HUMAN]	11.5
202	Haloacid dehalogenase-like hydrolase domain-containing protein 2 (Fragment) OS=Homo sapiens GN=HDHD2 PE=1 SV=1 - [K7ER15_HUMAN]	11.39
203	Ras-related protein R-Ras OS=Homo sapiens GN=RRAS PE=1 SV=1 - [RRAS_HUMAN]	11.33
204	14-3-3 protein zeta/delta OS=Homo sapiens GN=YWHAZ PE=1 SV=1 - [1433Z_HUMAN]	11.28
205	Mitotic checkpoint protein BUB3 (Fragment) OS=Homo sapiens GN=BUB3 PE=1 SV=1 - [J3QT28_HUMAN]	11.23
206	Ras suppressor protein 1 OS=Homo sapiens GN=RSU1 PE=1 SV=3 - [RSU1_HUMAN]	11.21
207	Sodium/potassium-transporting ATPase subunit alpha-1 OS=Homo sapiens GN=ATP1A1 PE=1 SV=1 - [AT1A1_HUMAN]	11.19
208	Probable ATP-dependent RNA helicase DDX17 OS=Homo sapiens GN=DDX17 PE=1 SV=2 - [DDX17_HUMAN]	11.15
209	Keratin, type II cuticular Hb3 OS=Homo sapiens GN=KRT83 PE=1 SV=2 - [KRT83_HUMAN]	11.09
210	Serpin B3 OS=Homo sapiens GN=SERPINB3 PE=1 SV=2 - [SPB3_HUMAN]	10.97
211	Guanine nucleotide-binding protein G(s) subunit alpha isoforms short OS=Homo sapiens GN=GNAS PE=1 SV=1 - [GNAS2_HUMAN]	10.87
212	Peptidyl-prolyl cis-trans isomerase OS=Homo sapiens GN=PPIA PE=1 SV=1 - [F8WE65_HUMAN]	10.85
213	Ras GTPase-activating-like protein IQGAP1 OS=Homo sapiens GN=IQGAP1 PE=1 SV=1 - [IQGA1_HUMAN]	10.85
214	Cathepsin G OS=Homo sapiens GN=CTSG PE=1 SV=2 - [CATG_HUMAN]	10.79
215	Very-long-chain enoyl-CoA reductase (Fragment) OS=Homo sapiens GN=TECR PE=1 SV=7 - [M0QXM3_HUMAN]	10.72
216	Destrin OS=Homo sapiens GN=DSTN PE=1 SV=1 - [F6RFD5_HUMAN]	10.57
217	Catalase OS=Homo sapiens GN=CAT PE=1 SV=3 - [CATA_HUMAN]	10.54

218	TIP41-like protein OS=Homo sapiens GN=TIPRL PE=1 SV=2 - [TIPRL_HUMAN]	10.48
219	Histone H3 OS=Homo sapiens GN=HIST2H3PS2 PE=1 SV=1 - [Q5TEC6_HUMAN]	10.23
220	Caspase-14 OS=Homo sapiens GN=CASP14 PE=1 SV=2 - [CASPE_HUMAN]	10.16
221	Serum deprivation-response protein OS=Homo sapiens GN=SDPR PE=1 SV=3 - [SDPR_HUMAN]	10.08
222	ATP-dependent 6-phosphofructokinase, liver type OS=Homo sapiens GN=PFKL PE=1 SV=6 - [PFKAL_HUMAN]	10.05
223	Cysteine-rich protein 2 (Fragment) OS=Homo sapiens GN=CRIP2 PE=1 SV=1 - [H0YFA4_HUMAN]	10
224	Calpain-1 catalytic subunit OS=Homo sapiens GN=CAPN1 PE=1 SV=1 - [CAN1_HUMAN]	9.92
225	60S ribosomal protein L18 OS=Homo sapiens GN=RPL18 PE=1 SV=1 - [G3V203_HUMAN]	9.86
226	S-formylglutathione hydrolase OS=Homo sapiens GN=ESD PE=1 SV=2 - [ESTD_HUMAN]	9.83
227	14-3-3 protein sigma OS=Homo sapiens GN=SFN PE=1 SV=1 - [1433S_HUMAN]	9.83
228	Gelsolin OS=Homo sapiens GN=GSN PE=1 SV=1 - [A0A0A0MS51_HUMAN]	9.81
229	Actin-related protein 2/3 complex subunit 1A OS=Homo sapiens GN=ARPC1A PE=1 SV=1 - [E9PF58_HUMAN]	9.74
230	Serpin H1 OS=Homo sapiens GN=SERPINH1 PE=1 SV=2 - [SERPH_HUMAN]	9.69
231	Dihydrolipoyllysine-residue succinyltransferase component of 2-oxoglutarate dehydrogenase complex, mitochondrial (Fragment) OS=Homo sapiens GN=DLST PE=1 SV=1 - [Q86SW4_HUMAN]	9.66
232	Voltage-dependent anion-selective channel protein 1 OS=Homo sapiens GN=VDAC1 PE=1 SV=2 - [VDAC1_HUMAN]	9.65
233	Alcohol dehydrogenase class-3 (Fragment) OS=Homo sapiens GN=ADH5 PE=1 SV=1 - [H0YAG8_HUMAN]	9.59
234	Heat shock protein beta-1 OS=Homo sapiens GN=HSPB1 PE=1 SV=2 - [HSPB1_HUMAN]	9.59
235	Protein disulfide-isomerase A3 OS=Homo sapiens GN=PDIA3 PE=1 SV=4 - [PDIA3_HUMAN]	9.59
236	Guanine nucleotide-binding protein G(I)/G(S)/G(T) subunit beta-1 (Fragment) OS=Homo sapiens GN=GNB1 PE=1 SV=7 - [B1AKQ8_HUMAN]	9.47
237	Ras-related C3 botulinum toxin substrate 1 OS=Homo sapiens GN=RAC1 PE=1 SV=1 - [RAC1_HUMAN]	9.38
238	Zinc-alpha-2-glycoprotein OS=Homo sapiens GN=AZGP1 PE=1 SV=2 - [ZA2G_HUMAN]	9.33
239	Ribose-phosphate pyrophosphokinase 2 OS=Homo sapiens GN=PRPS2 PE=1 SV=2 - [PRPS2_HUMAN]	9.31
240	Adenylyl cyclase-associated protein 1 OS=Homo sapiens GN=CAP1 PE=1 SV=5 - [CAP1_HUMAN]	9.23
241	tRNA-splicing ligase RtcB homolog OS=Homo sapiens GN=RTCB PE=1 SV=1 - [RTCB_HUMAN]	9.21
242	Protein argonaute-2 OS=Homo sapiens GN=AGO2 PE=1 SV=3 - [AGO2_HUMAN]	9.21
243	Carbonic anhydrase 1 (Fragment) OS=Homo sapiens GN=CA1 PE=1 SV=1 - [E5RIF9_HUMAN]	9.17
