# Supplementary material for: Oncogenic TRIB2 interacts with and regulates PKM2 to promote aerobic glycolysis and lung cancer cell procession
Source: Cell Death Discov. 2022 Jul 5;8:306. doi: 10.1038/s41420-022-01095-1 (PMC9256704; doi:10.1038/s41420-022-01095-1)
Supplement: Supplementary file 3 — author contributions [file 41420_2022_1095_MOESM3_ESM.docx]

**AUTHORS’ CONTRIBUTIONS**

S.Y.X. conceived the study; P.Y.W. and S.Y.X. designed experiments; Y.R.L., D.D.S. and R.R.W. performed FACS experiments; Y.R.L., D.D.S. M.L.Z., and D.M.L. performed immunofluorescence staining, RT-PCR, immunoprecipitation and immunoblotting and the aerobic glycolysis experiments; Y.J.L. and Z.Y. performed immunohistochemistry staining; Y.F.Y., H.F.S., and J.X.H. produced mouse model and HE staining; Y.M.L. collected samples; Y.L.Z. and Y.L. performed the experiments of cell proliferation, cell colony, and cell migration; Y.L. and H.F.Z analyzed the data; P.Y.W. analyzed the experiments with human samples; S.Y.X. and P.Y.W. prepared figures; Y.F.Y. and S.Y.X. wrote the manuscript. All authors read and approved the final version of the manuscript.
