# Supplementary material for: Oncogenic TRIB2 interacts with and regulates PKM2 to promote aerobic glycolysis and lung cancer cell procession
Source: Cell Death Discov. 2022 Jul 5;8:306. doi: 10.1038/s41420-022-01095-1 (PMC9256704; doi:10.1038/s41420-022-01095-1)
Supplement: Supplementary file 4 — animal Ethics [file 41420_2022_1095_MOESM4_ESM.pdf]

# 滨州医学院动物伦理委员会

The Animal Ethics Committee of Binzhou Medical University

## 涉及动物研究项目伦理审批件

伦研批第（ 2017-02-02）号

| | | | |
|---|---|---|---|
| 项目名称 Project | Oncogenic TRIB2 interacts with and regulates PKM2 to promote aerobic glycolysis and lung cancer cell procession | | |
| 项目类别 (Samples) | Animals | | |
| 项目负责人 | Shu-Yang Xie | 职　　称 | Professor |
| 项目承担单位 | Binzhou Medical University | 主要负责人 | Shu-Yang Xie |
| 伦理审查意见 | | | |
| Δ同意 Approve | √ | | |
| Δ不同意（项目终止或暂停）Disapprove | | | |

审批意见

All animal care and experiments were performed in accordance with the Guidelines for Care and Use of Laboratory Animals of National Institutes of Health guidelines and approved by the Committee on the Ethics of Animal Experiments of Binzhou Medical University. The protocol as follows: A549 cells treated with lentivirus stably expressing shRNA-TRIB2, siRNA-PKM2, TRIB2-expression or PKM2-expression, are harvested. $2 \times 10^6$ cells are injected subcutaneously into the backs of BALB/C-nu nude mice aged 6–8 weeks (HFK Bio-Technology, Beijing, China). Then, primary tumors are measured daily by a caliper. One month later, the animals will be euthanized by intraperitoneal injection of a barbiturate. The bone marrow cells from TRIB2-/- and wild type mice will be cultured to investigate glucose uptake, lactate production, and ATP content.

经动物伦理委员会讨论，认为该研究项目所涉及的研究内容、范围和研究方法符合医学伦理的相关要求，同意开展研究。

滨州医学院动物伦理委员会

The Animal Ethics Committee of Binzhou Medical University

（盖章）

2017 年 02 月 02 日
