# Supplementary material for: Oncogenic TRIB2 interacts with and regulates PKM2 to promote aerobic glycolysis and lung cancer cell procession
Source: Cell Death Discov. 2022 Jul 5;8:306. doi: 10.1038/s41420-022-01095-1 (PMC9256704; doi:10.1038/s41420-022-01095-1)
Supplement: Supplementary file 5 — Medical Ethics [file 41420_2022_1095_MOESM5_ESM.pdf]

滨州医学院医学伦理委员会

The Medical Ethics Committee of Binzhou Medical University

涉及人的生物医学研究项目伦理审批件

伦研批第（ 2018-02-20）号

项目名称 Project	Oncogenic TRIB2 interacts with and regulates PKM2 to promote aerobic glycolysis and lung cancer cell procession		
项目类别 (Samples)	Human cancer tissues		
项目负责人	Shu-Yang Xie	职　　称	Professor
项目承担单位	Binzhou Medical University	主要负责人	Shu-Yang Xie
伦理审查意见			
Δ同意 Approve		√	
Δ不同意（项目终止或暂停）Disapprove			

审批意见

All experiments were approved and performed according to the Medical Ethics Committee of Binzhou Medical University. Sectioned NSCLC tissues will be collected between July 1, 2018 and December 30, 2020 from the Inpatient Department of Chest surgery, Yantai Shan Hospital, the Teaching Hospital of Binzhou Medical University (Yantai, China). Patients pathologically diagnosed with NSCLC for the first time and had not yet received chemotherapy, will be included in the present study. Fresh NSCLC tissues and para-carcinoma controls from the patients who underwent surgery arestudied. The levels of p-PKM2 (ser37)/PKM2 and TRIB2 are analyzed in the tissues to investigate their roles in lung cancer. The study procedures are fully explained to patients before study inclusion, and patients will be provided written informed consent.

经医学伦理委员会讨论，认为该研究项目所涉及的研究内容、范围和研究方法符合医学伦理的相关要求，同意开展研究。

滨州医学院医学伦理委员会

The Medical Ethics Committee of Binzhou Medical University

（盖章）

2018 年 02 月 20 日
